# Perfusion double-channel micropipette probes for oxygen flux mapping with single-cell resolution

**DOI:** 10.3762/bjnano.9.79

**Published:** 2018-03-09

**Authors:** Yang Gao, Bin Li, Riju Singhal, Adam Fontecchio, Ben Pelleg, Zulfiya Orynbayeva, Yury Gogotsi, Gary Friedman

**Affiliations:** 1 Department of Electrical and Computer Engineering, Drexel University, 3141 Chestnut Street, Philadelphia, PA 19104, USA; 2 Department of Material Science and Engineering, Drexel University, 3141 Chestnut Street, Philadelphia, PA 19104, USA; 3 Department of Surgery, Drexel University, 245 N. 15th Street, Philadelphia, PA 19102, USA

**Keywords:** double-barrel pipette, hydrodynamic confinement, perfusion, oxygen flux, single-cell metabolic analysis

## Abstract

Measuring cellular respiration with single-cell spatial resolution is a significant challenge, even with modern tools and techniques. Here, a double-channel micropipette is proposed and investigated as a probe to achieve this goal by sampling fluid near the point of interest. A finite element model (FEM) of this perfusion probe is validated by comparing simulation results with experimental results of hydrodynamically confined fluorescent molecule diffusion. The FEM is then used to investigate the dependence of the oxygen concentration variation and the measurement signal on system parameters, including the pipette’s shape, perfusion velocity, position of the oxygen sensors within the pipette, and proximity of the pipette to the substrate. The work demonstrates that the use of perfusion double-barrel micropipette probes enables the detection of oxygen consumption signals with micrometer spatial resolution, while amplifying the signal, as compared to sensors without the perfusion system. In certain flow velocity ranges (depending on pipette geometry and configuration), the perfusion flow increases oxygen concentration gradients formed due to cellular oxygen consumption. An optimal perfusion velocity for respiratory measurements on single cells can be determined for different system parameters (e.g., proximity of the pipette to the substrate). The optimum perfusion velocities calculated in this paper range from 1.9 to 12.5 μm/s. Finally, the FEM model is used to show that the spatial resolution of the probe may be varied by adjusting the pipette tip diameter, which may allow oxygen consumption mapping of cells within tissue, as well as individual cells at subcellular resolution.

## Introduction

Transport, production and consumption of gasses, ions, and organic molecules are fluxes that sustain life. Relatively few tools are available to control and map these fluxes at the microscopic scale commensurate with the size of individual cells. While there has been progress in obtaining snapshots of genomic and transcriptomic information from single cells [1–3], the lack of microscopic tools that measure and control fluxes limits studies of metabolic variability of cells within cell populations. Measurements of single-cell metabolic rates are important, as it has been shown that even genetically identical cells can behave differently [4]. The use of molecular or nanoparticle fluorescent reporters is a well-developed technique for imaging of concentrations of various molecular and ionic species in cells and tissues [5–6], but non-uniformities in the natural distribution of fluorescent reporters limits their applications in assessing fluxes due to individual cells. Furthermore, concerns often exist regarding potential toxicity of exogenous fluorescent agents [7]. An alternative approach is to map concentration gradients using scanning probes that may employ some means of sensing such as electrochemical or optical [8–15]. These types of probes can attain subcellular scale resolution when their tip size is smaller than the size of a cell [10–11]. However, the sensitivity of most sensors is typically proportional to their effective area, so sensors with relatively higher spatial resolution have lower sensitivity [14–16], or require a drastically increased measurement time. Here we propose and investigate a scanning flux measurement system for individual cells that offers high sensitivity and high spatial resolution. The main concept of the developed scanning probe is the confinement of the flux being measured by use of flow perfusion through double-channel micropipettes.

We specifically focus on the measurement of oxygen consumption by individual cells as a case study, although various other types of functional analyses are possible [12,17]. Since the time of the 1931 Nobel Prize winning work of Otto Warburg [18], respirometry has been widely employed to characterize metabolism and mitochondrial functions of cell cultures, tissues and larger organisms [19–23]. Commercially available respirometry tools that are capable of carrying out measurements on about 10^5^–10^6^ cells typically rely on sealing cells within oxygen tight chambers while measuring reduction of oxygen concentration over time as various sequences of mitochondrial modulators and substrates are added to the cell suspension [24–25].

Recent work using oxygen sensing, based on quenching of luminescence due to oxygen, has demonstrated the capability to carry out respirometry on single cells in sealed microchambers [26–28]. However, the primary difficulty with sealed chamber approaches is maintaining control over the cellular environment during an experimental time scale longer than tens of minutes. Maintaining a relatively constant carbon dioxide concentration, oxygen concentration, pH and nutrient supply requires using relatively large amounts of extracellular fluid per cell (typically few millions of cells per 1 mL [29]), reducing the sensitivity to oxygen concentration variations. Electrochemical scanning probes have been used to measure oxygen concentration variations near cells due to their respiration [30] and can be made with tips smaller than 100 nm in diameter [9,31–32]. However, no clear relationship between oxygen consumption and oxygen concentration near the cells has been obtained [30,33]. Another alternative that has been considered for measuring respiration of embryos and oocytes is to employ a system where linear oxygen gradients are measured by moving a sensor along a small tube with the embryo (a group of cells) at one end [34]. This technique demonstrated the ability to measure respiration rates of around 0.7 fmol/s, which is 100–1000 times faster than typical oxygen consumption rates of small individual cells.

**Figure 1:**
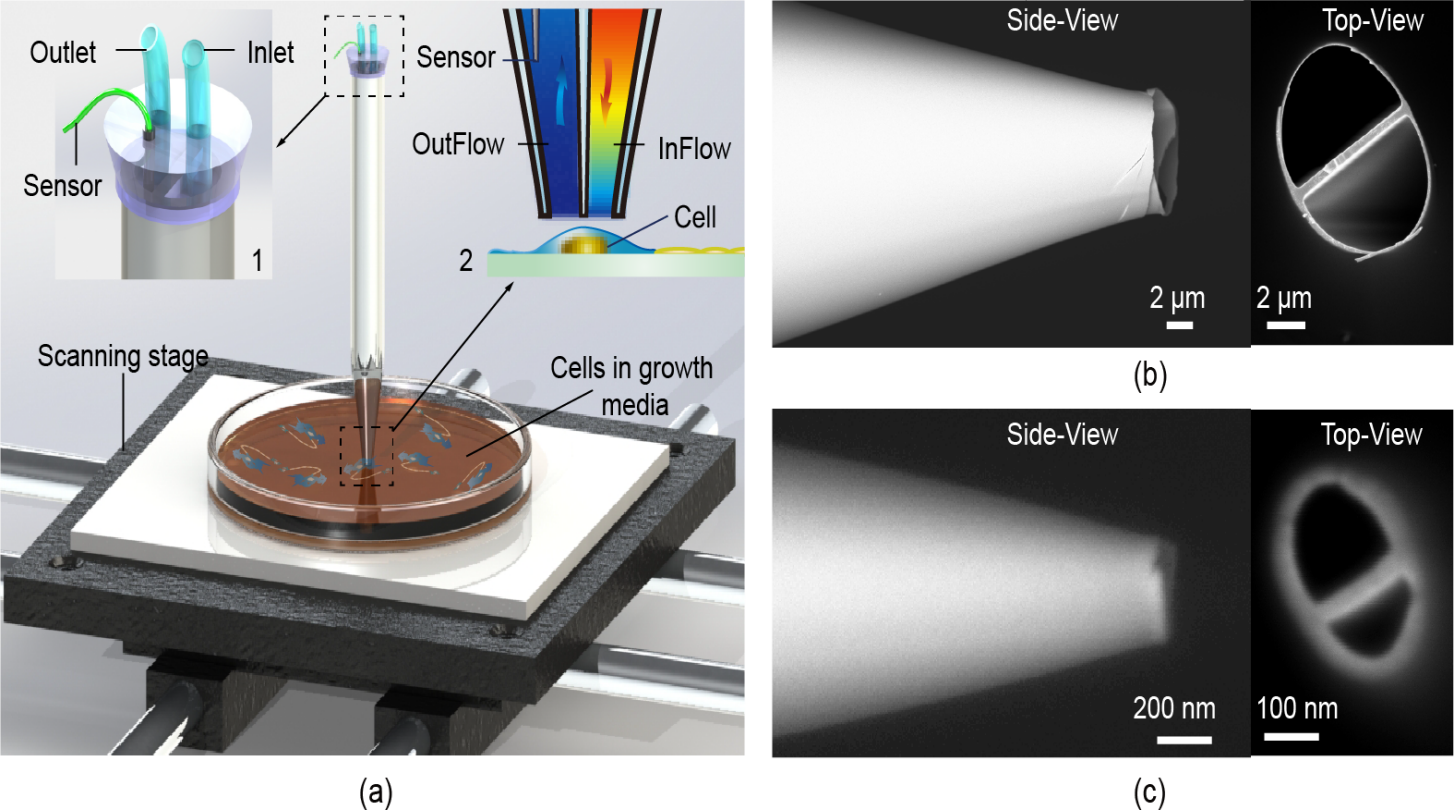
a) Illustration of the double-barrel perfusion-based single-cell respirometry probe. The cell culture or tissue dish is shown on top of an *x*–*y*–*z* positioning set-up. The inset (1) shows tubing for the inlet and outlet channels in both channels as well as a sensor in the outlet channel. The inset (2) illustrates the differences in oxygen concentration upstream and downstream from the cell within the theta pipette. The different colors represent different oxygen concentrations that are obtained from a finite element simulation of convection–diffusion equations. Inset (2) also illustrates that an oxygen sensor positioned downstream from the cell can be used to determine the cell’s oxygen consumption rate when the sensor’s measurement refers to the oxygen concentration at the top of the theta pipette. b) SEM images of a micrometer-scale theta pipette, side-view and top-view. The tip width (approximately representing the tip diameter (dw) parameter used in the simulation model) measured from the outer wall in the side-view image is 8 μm. c) SEM images of a nanometer-scale theta pipette, side-view and top-view. The tip width measured from the outer wall in the side-view image is 300 nm.

The general idea behind the proposed use of a double-channel pipette for oxygen consumption measurement by individual cells in a cell culture is illustrated in Figure 1a. The SEM images of two theta pipettes (whose cross-sections looks like the Greek letter θ, where the top and bottom opening are associated with different channels) with tip diameters (dw) of 8 μm and 300 nm are shown in Figure 1b,c. Although the theta pipette is one type of double-channel pipette, there are other types, such as those with concentric channels [35] that can be manufactured and used. The key function of the pipette is to confine the oxygen flux between its two ends, reducing the lateral spread of the oxygen molecules being detected, while permitting the use of sensors with larger effective areas positioned further away from source of flux. The focus of this paper is on investigating the effects of various system parameters such as the half-angle of a theta pipette, position of the oxygen sensors within the pipette, perfusion flow rate and distance of the pipette tip from the substrate on oxygen flux sensitivity. The effects of varying the aforementioned parameters will be studied below using a finite element model (FEM) of the double-barrel pipette with perfusion. To validate this model, we first compare hydrodynamic confinement obtained from the model with experiments using a fluorescent dye. Later in the paper, we also show that FEM results agree qualitatively with a simplified analytical model.

## Results and Discussion

### Hydrodynamic confinement

Consider flow within a long channel: a molecule cannot diffuse outside the channel due to the presence of hard channel walls. However, if the channel walls are missing along some length segment of the flow, the molecule may diffuse outside the channel, unless the flow velocity is high enough that the molecule moves through this section before it has a chance to diffuse through the gap. Therefore, in the section where the channel walls are missing, like the section at the tip of the double-barrel pipette, the molecule could remain hydrodynamically confined to the flow. The time that it takes a molecule to diffuse across the section of length *b* along the flow is *b*^2^/*D*, where *D* is the diffusion coefficient, and the time it takes the flow to cross the same distance is roughly *b*/*v*, where *v* is the flow velocity. Taking the ratio of these times, we obtain the Peclet number Pe = *bv*/*D*, which indicates the relative importance of convective transport (flow) over the diffusion. When the Peclet number is large, the diffusion time is larger than convective transport time and the likelihood that a molecule remains confined in the flow is high. This simple idea of hydrodynamic confinement has been discussed in the microfluidics literature [28,36] and in some biological applications [33,37–38], some of which employed a concentric double-channel pipette [38].

**Figure 2:**
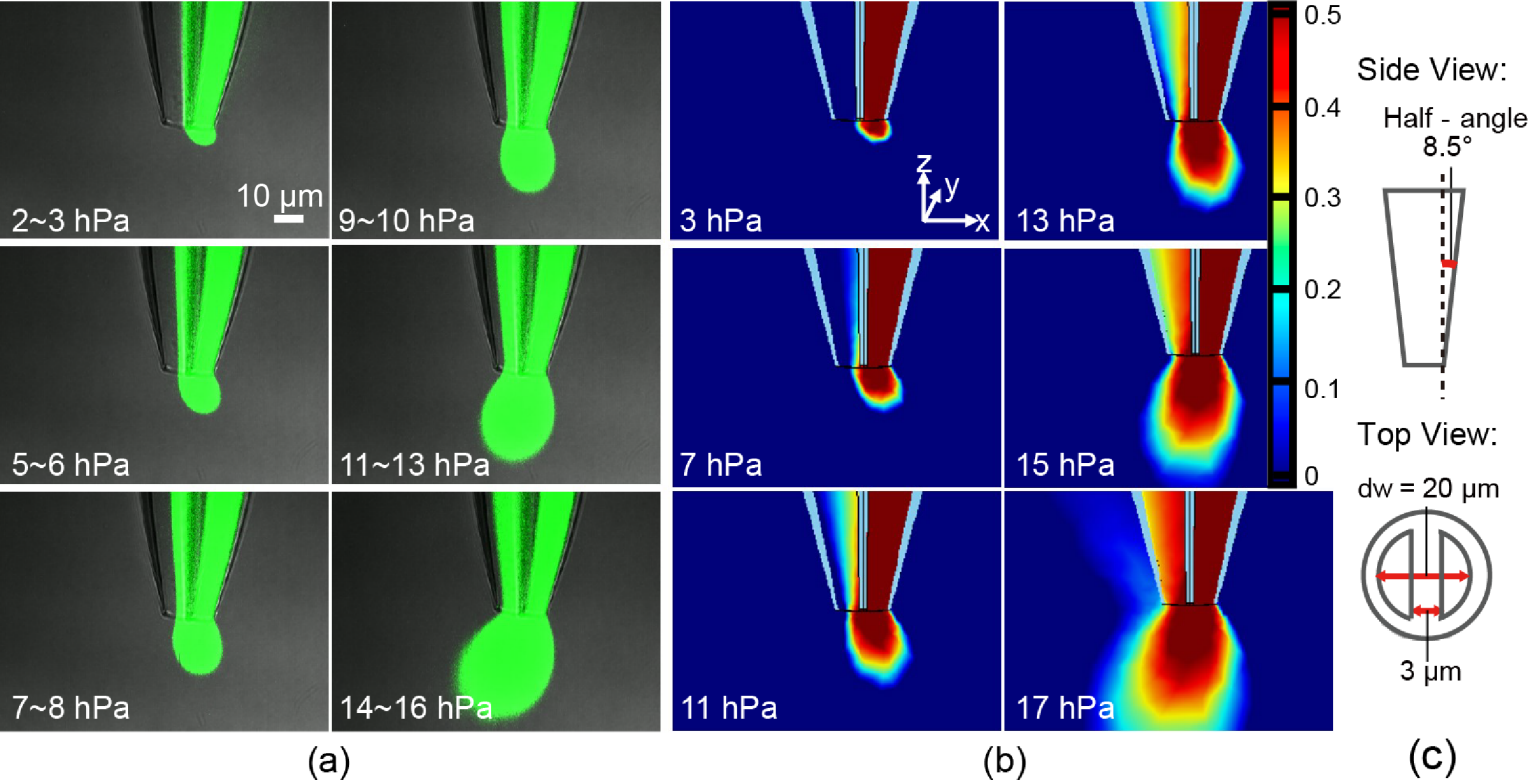
Comparison between experimental and simulated perfusion flow patterns. a) Microscope images of flow patterns. The injecting flow is a saturated solution (0.08 wt %) of fluorescein. Images 1–6 show patterns at increasing injection pressure, while the extraction flow rate is held constant (5 µL/min). The left channel is the extraction channel, and the right channel is the pressurized injection channel, *P*_in_ = *P*_applied_total_pressure_ − 1 atm, 1 hPa = 100 Pa. b) Simulated perfusion flow patterns. The geometrical parameters to define the pipette were obtained by measurements from optical microscope images in Figure 2a (septum thickness, side wall half-angle, etc.) and similar pressure/suction settings were used as in the experiment. c) Geometrical sketches of Figure 2a and Figure 2b, side view (top) and cross-section (bottom) view.

Here, we report experimental observation of hydrodynamic confinement at the tip of the theta pipette and compare it with a finite element method (FEM) model that implements both Navier–Stokes equations to model the fluid flow and convection–diffusion equations to model molecular diffusion (see Experimental section for a detailed discussion). In the experiment, the fluid is being withdrawn at a fixed rate of 5 µL/min through one channel, while pressures from 2 to 16 hPa are applied to the injection channel. Experimentally observed diffusion of the fluorescent dye for different injection pressures is shown in Figure 2a. Two qualitative trends can be noted. One is the increasing size of the fluorescent plume with the increase in the pressure applied to the injection channel. It is clear from the images that at low pressure (≈2–3 hPa), the plume is smaller than the pipette tip, while at higher pressure (15–17 hPa), the plume is larger than the pipette tip. The other trend is the change in the tilt of the diffusion plume with increasing pressure applied to the injection channel. At lower injection pressures, the plume shape is dominated by the existing flow field near the tip, which develops due to the strong suction exerted by the extraction channel to support the applied flow rate and appears tilted away from the extraction channel. At higher injection pressures, the injection channel is able to contribute more fluid to the extraction channel. Thus, the suction exerted by the extraction channel is reduced and the resulting flow field near the tip (and the plume shape) starts to evolve and tilt more towards the extraction channel. Therefore, the average dye concentration in the extraction channel also increases as the injection pressure is increased. Figure 2b shows the results of the FEM where the pipette diameter at the tip and other geometrical parameters of the pipette were similar to the experimental parameters. The flow rate in the extraction channel in the simulations was set equal to the experimental flow rate, and the pressure through the injection channel (in the simulation) was adjusted until the diffusion plume size and shape matched what was experimentally observed. It can be seen that there is close agreement between injection pressures in the FEM model and the experimental observations for any given size and shape of the plume, suggesting that the model is valid over this range of conditions.

### Oxygen confinement due to pipette and effect of increased pipette diameter

**Figure 3:**
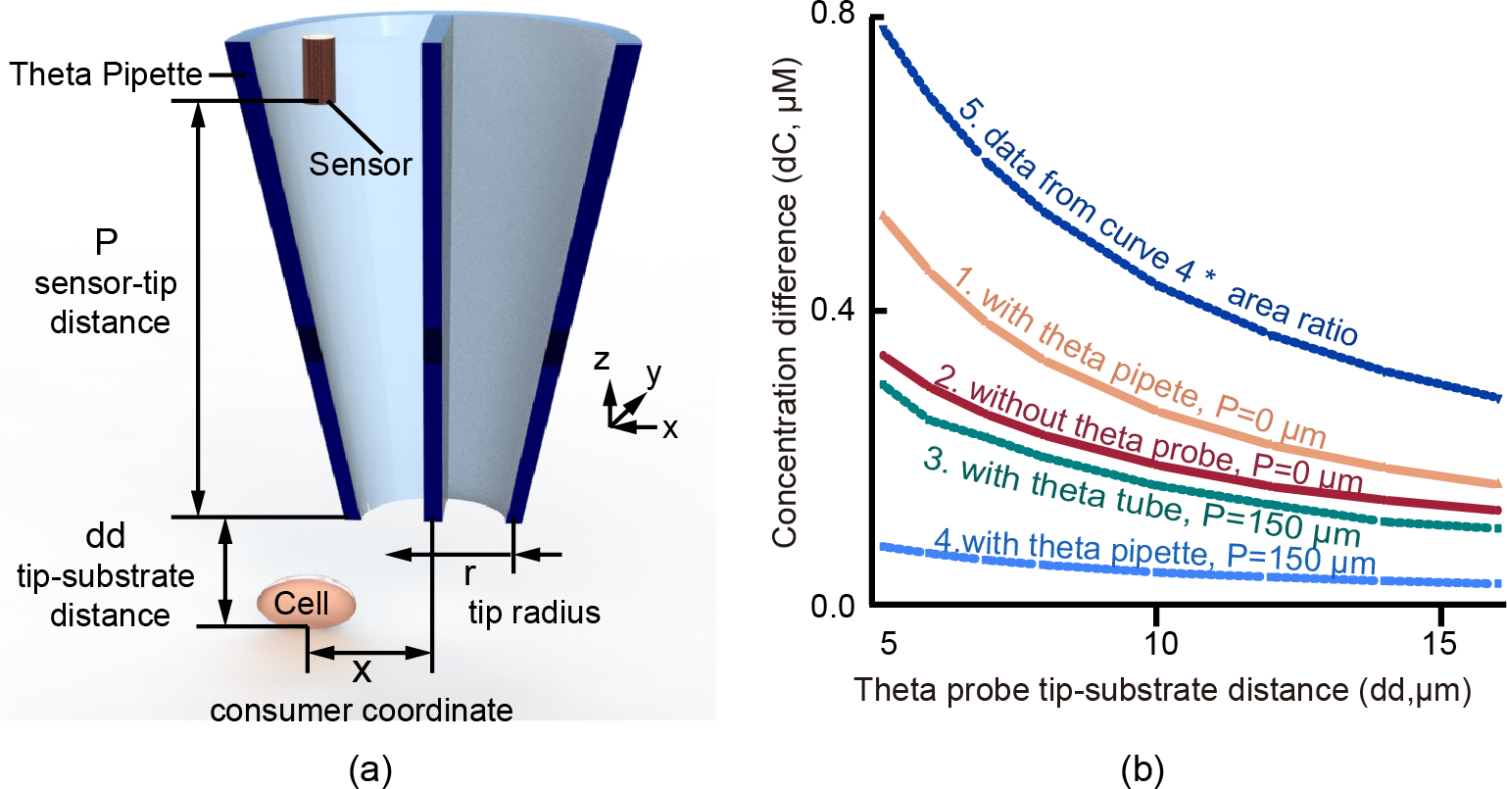
a) Demonstration of the simulation geometry parameters. Details of the parameters used in the model are specified in the Experimental section. b) Dependence of signal (oxygen concentration difference) strength on the proximity of the theta pipette tip to the substrate, for two sensor locations, and two pipette half-angles (with the cell noted as the consumer beneath the pipette, and without flow between barrels). Curve 1 is plotted from a sensor located at the tip of a typical theta pipette which has a half-angle of 8.5° and a tip diameter of 20 μm; Curve 2 is from a sensor at the same location, but without a theta pipette surrounding it; Curve 3 is from a sensor located inside a theta tube (0° half-angle, and tip diameter of 20 μm) and 150 μm above the tip; Curve 4 is a plot from the sensor located inside a typical theta pipette and 150 μm above its tip; Curve 5 is curve 4 multiplied by the area ratio of this location and the tip.

One influence of the pipette is the confinement of oxygen diffusion within it. Oxygen can diffuse freely along the pipette axis (*z*-axis as in Figure 3a), but remains confined by the pipette walls. To demonstrate the effect of this confined diffusion, consider a small oxygen sensor positioned at a small distance from the cell. The geometry parameters used in the model are demonstrated in Figure 3a. As demonstrated by FEM simulations results shown in Figure 3b (curve 1 vs 2), the oxygen concentration difference signal (the difference between the concentration of a saturated oxygen solution in water at room temperature and the oxygen concentration at the specific point under investigation) obtained by the sensor positioned at the tip of the pipette has around 1.5 times greater signal than the same size sensor placed at the same distance away from the cell, but without the pipette. This is because of the proximity of the pipette to the cell results in an oxygen concentration gradient within the pipette due to oxygen consumption by the cell. The oxygen gradient within the pipette is larger than the gradient of oxygen in the surrounding fluid because oxygen is constrained within the pipette to diffuse effectively only along the pipette length. This results in oxygen diffusing slower within the pipette than in the surrounding fluid. Thus, the mere presence of the pipette over the cell increases the oxygen concentration difference that can be sensed.

Figure 3b also shows that moving the same sensor within the pipette much further from the cell, while maintaining the same distance of the pipette tip from the cell, reduces the signal, as might be expected. Most of this reduction can be attributed to the expansion of the pipette diameter away from the cell due to a non-zero pipette half-angle. This conclusion can be confirmed by considering a theta pipette with a zero half-angle (theta tube, curve 3 in Figure 3b). As demonstrated in Figure 3b, the signal obtained by a sensor placed 150 μm away from the tip of the theta tube is nearly the same as the signal obtained by placing the sensor close to the cell without the tube (curve 2 in Figure 3b).

So far, we have considered sensors that remain the same in size regardless of their position along the axis of the theta pipette. However, considering that the diameter of the pipette increases away from the tip (for non-zero half-angles), sensors that are larger in size can be facilitated. If we scale the sensor area with the increasing pipette diameter, the signal can be improved significantly depending on the nature of the sensor. For example, the electrical current used as the signal in electrochemical sensors is proportional to the effective sensor area. If we take the sensor sensitivity to be proportional to the area, we can significantly increase the overall sensitivity of the probe as we move the sensor further away from the pipette tip, which is also indicated in Figure 3b (curve 5 vs 1). Therefore, this analysis suggests an opportunity to improve sensitivity without sacrificing resolution. One may wonder why the signal strength is increased when the concentration decreases in a pipette whose cross-sectional area increases along its *z*-axis (as in Figure 3a). Diffusion along a non-zero half-angle pipette, whose diameter increases along its axis proportional to the axial distance, can be modeled as diffusion in a solid angle of a sphere. Such a model would yield a concentration that decreases linearly with the axial distance. At the same time, the sensor area would increase as the square of the axial distance, resulting in a linear gain of sensitivity with distance for a sensor whose sensitivity is proportional to its area.

### Effects of perfusion on oxygen consumption signaling

**Figure 4:**
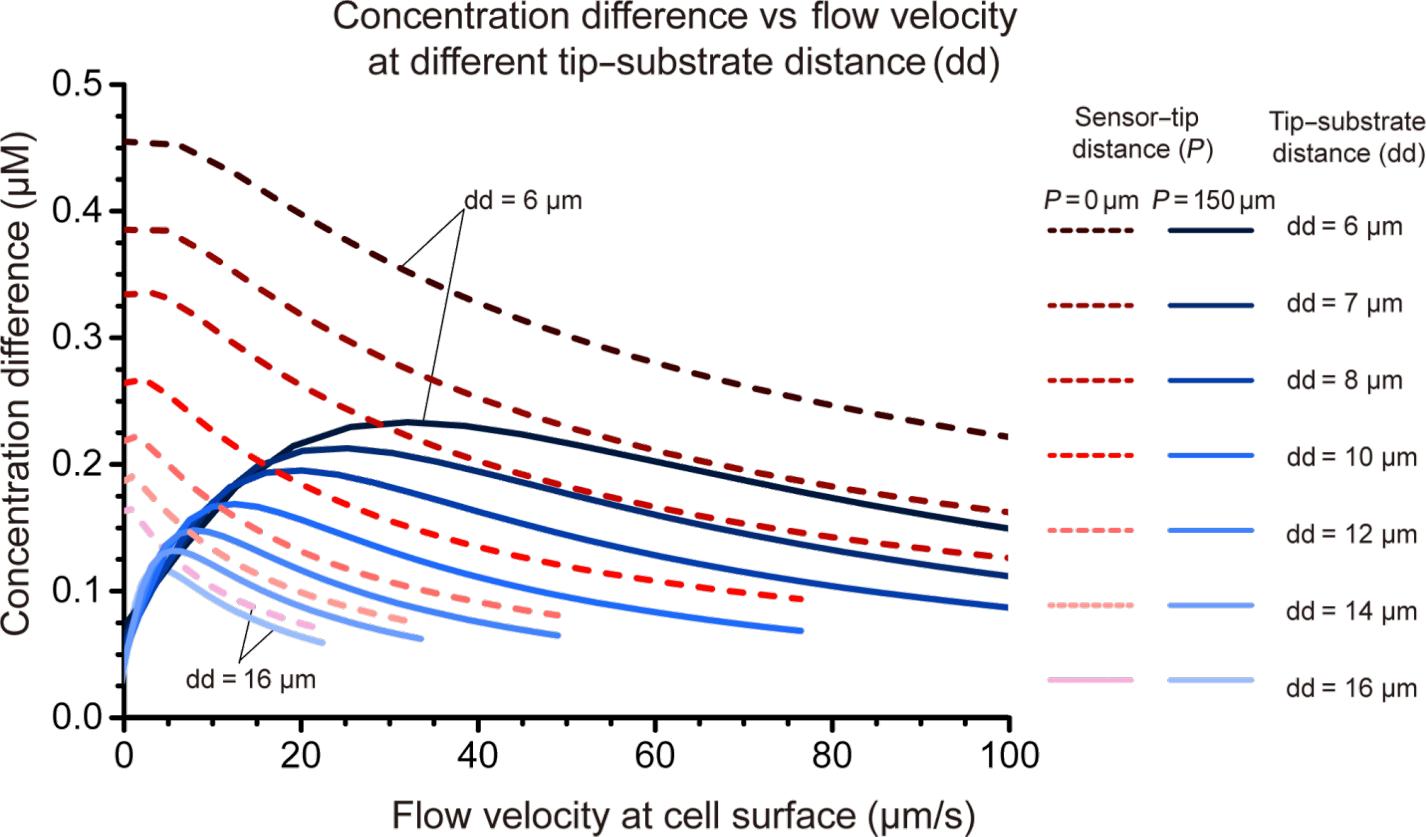
Dependence of signal (oxygen concentration difference) strength on perfusion flow velocity for different tip–substrate distances (dd), and for sensors at different locations (0 μm and 150 μm above the tip) inside a typical theta pipette (tip diameter dw = 20 μm, half-angle = 8.5°). Other parameter settings are the same as those used for Figure 3. The group of red dotted curves are measured from the sensors at the tip of the theta pipette. The group of blue solid curves are measurements from sensors inside the theta pipette, 150 μm above the tip. For both color groups, from dark color to light color, the distance from the theta pipette tip to substrate increases.

One may expect that losing less molecules to the diffusion away from the sensor should increase the probe sensitivity. As shown in Figure 4, this effect is indeed confirmed by the FEM calculations when considering a sensor placed 150 μm downstream within the theta pipette. One would expect significant amplification of the signal (oxygen concentration difference) due to perfusion to occur when diffusion dominates over the convection and the Peclet number is significantly smaller than 1, say 0.1. In such operating regime, all oxygen molecules are not fully retained within the flow, and increases in the flow velocity help to retain oxygen molecules. At larger flow velocities, most oxygen molecules are already confined to the flow and further velocity increases do not amplify the signal. This logic can provide a rough estimate of the perfusion velocity range beyond which no signal is gained. As an example, we consider a pipette that is located at dd = 10 μm from the substrate. Taking the oxygen diffusion coefficient of 2000 µm^2^/s and assuming that amplification occurs mostly below the Peclet number of 0.1, one finds that no significant signal gain should occur beyond the velocity *v*_max_* ≈* Pe*·D*/dd = (0.1 × 2000)/10 = 20 μm/s. This is in quantitative agreement with the velocity of maximal signal calculated by the FEM and shown in Figure 4.

As the velocity increases further, the flux of oxygen in the pipette due to flow should start dominating the flux due to oxygen consumption, reducing the oxygen concentration difference along the *z*-axis of the extraction channel. The reduction of oxygen concentration difference between different positions along the flow at higher flow velocities can be demonstrated by a simplified analytical model (see Supporting Information File 1) and is given by Equation 1:

[1]
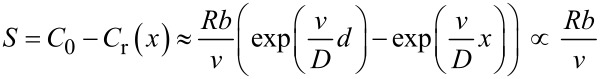


where *R* is the oxygen consumption rate per unit length of the flow, *b* is the length of the oxygen consumption region in the flow, *d >> b* is the distance from the tip of the pipette to the place in the flow where a constant oxygen concentration, *C*_o_, exists due to contact with the environment, *x* is the position of the sensor downstream from the consumption region and *C*_r_(*x*) is the oxygen concentration measured by the sensor.

As shown in Figure 4, calculated based on a normal human prostate cell oxygen consumption rate (10^−17^ mol/s), an oxygen concentration difference of 0.27 μM could be measured by the sensor at *P* = 150 μm under optimized flow conditions. This concentration difference is close to commercial oxygen optical probe resolution with a similar sensing area (from data sheet of fiber optic oxygen sensor from Pyroscience, with a tip size of 35 μm in diameter and a resolution of 0.78 μM at 20% oxygen). In a real case scenario, we can assume the oxygen consumption rate of a tumor cell is 10 times higher than that of a normal cell [39], which yields a resolution of 2.7 μM with the designed sensor.

### Spatial resolution

**Figure 5:**
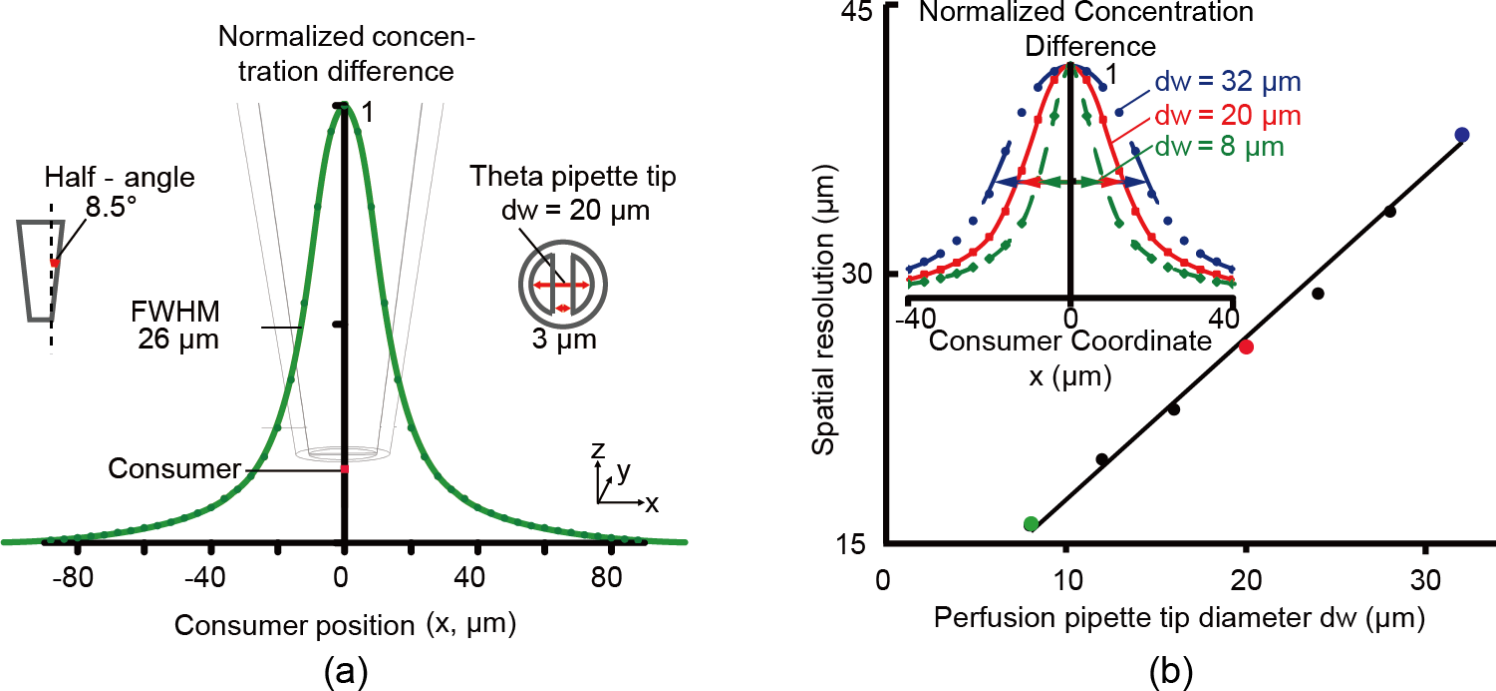
Spatial resolution of the scanning theta pipette probe. a) Illustration of the spatial resolution of a perfusion respirometry probe with tip diameter (dw) of 20 µm. The probe is located at the center of the *x*-axis and the specific tip geometry (as in insets) is the same as in the previous simulations. The oxygen concentration difference is the desired signal and is measured by the sensor inside the probe, 150 µm above the tip. A tiny, cubic consumer element of volume 2 × 2 × 2 µm^3^ is located on the substrate. The distance from substrate to theta pipette tip is set at 6 µm. The pressure applied at the input channel is (1 atm + 2 Pa), and at the output is (1 atm − 2 Pa). With these pressure values, the impact of perfusion on improving the signal is most significant. The green curve is a plot of the measured signals as the center position of the consumer moves from left (*x* = −90 µm) to right (*x* = 90 µm) along the *x*-axis. The full width at half maximum (FWHM) of this curve is 26 µm, and can be defined as the spatial resolution. b) The probe spatial resolution vs tip diameter. The pressure at the inlet and outlet of the theta pipette is set to achieve best improvement of signal at 150 µm above the tip for each tip diameter, respectively. The theta pipette tip diameter increases from 8 to 32 µm with a step size of 4 µm, while the half-angle of the pipette is fixed (8.5°). Inset: the oxygen concentration difference plot at tip sizes of 8, 20 and 32 µm.

One important role of the theta pipette probe is to increase sensitivity by placing sensor further up the pipette and using the perfusion flow, while preserving spatial resolution to permit measurements from individual cells in cell culture. Figure 5 illustrates that high resolution is achievable. In fact, it shows that the resolution is on the order of the pipette diameter and, since diameters smaller than micrometers are readily achievable [40–42], spatial resolution on the order of micrometers is possible.

## Conclusion

This paper studies a perfusion double-barrel micropipette, in particular, a theta pipette, as a microfluidic system that is potentially important for investigating metabolic variations among individual cells associated with changes in biological functions and disease development. The use of FEM to study the behavior of this microfluidic system not only verifies the experimental results, demonstrating the feasibility of the proposed approach, but also allows theoretical insights into pipette performance/sensitivity to be obtained that would otherwise require extensive studies, if done experimentally. In particular, the effects of the theta micropipette operational parameters on the system oxygen sensing capacity were considered first. It was found that the mere presence of the pipette over the cell increases the oxygen concentration difference that can be sensed. Also, the use of the theta pipette increases the overall sensitivity of the probe as the sensor is moved away from the pipette tip, due to oxygen confinement. In addition, the larger diameter of the pipette channel far from the tip allows the use of sensors with larger surface area. When the sensor is placed far from the tip end, introducing an appropriate perfusion flow to the system not only maintains a constant cell microenvironment, but also further confines the free diffusion, amplifying the signal (oxygen concentration difference) at the sensor location and preventing back diffusion. Finally, in this paper we focused on theta pipettes with micrometer-scale tips (Figure 1b), which would cover an average cell area to maximize the signal intensity. However, pipettes with sub-micrometer tips (Figure 1c) could be produced to obtain spatial resolution at subcellular levels. It is also worth mentioning that the developed probe is certainly not limited to oxygen measurement. By using different sensors, including electrochemical or optical ones, other types of analyses can be carried out over the surface of living tissue. One can also envision applications of the proposed approach in analytical chemistry or forensic study for spatially resolved microanalysis.

## Experimental

### Description of experimental set-up for experimental hydrodynamic confinement

**Figure 6:**
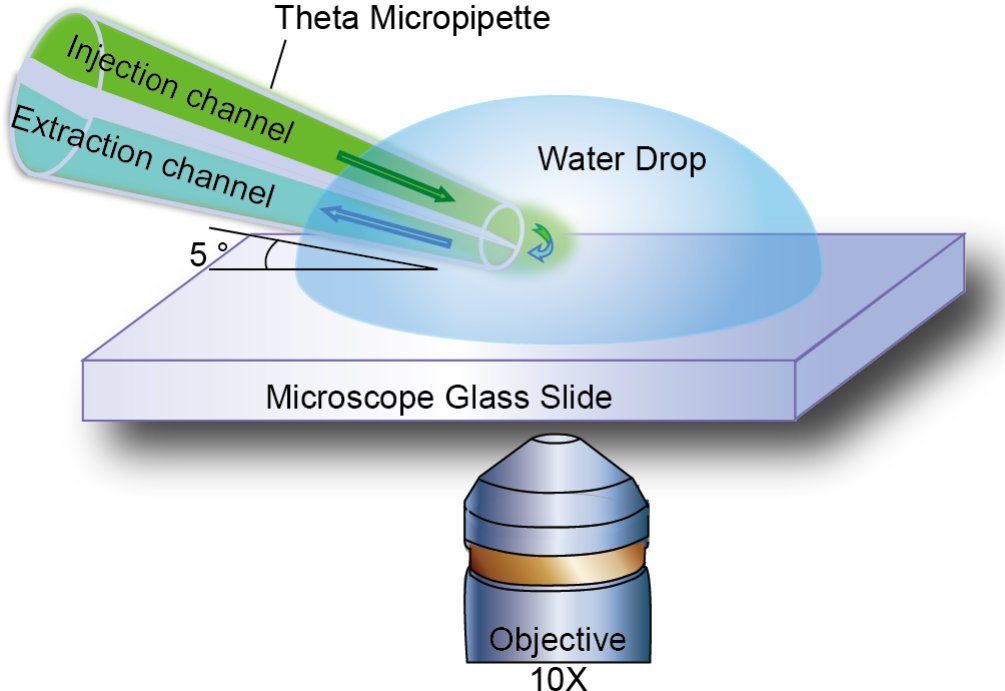
Illustration of the experimental setup for investigating the effect of perfusion flow on the diffusion of fluorescent dye. The blue hemisphere is the water drop (0.3 mL) formed over a microscope glass slide. The theta pipette tip is inserted into the water drop. The injection channel is preloaded with a saturated fluorescein aqueous solution (0.08 wt %). The green color represents the fluorescein dye (fluorescein), with the green intensity proportional to the dye concentration. The small green area volume within the water drop represents roughly the diffusion boundary of the florescent dye. The arrows show the fluid flow direction. A constant withdraw rate of 5 µL/min is applied by a syringe pump to the pipette’s extraction channel. The injection pressure was increased from 100 hPa to 116 hPa with an increment of 2–3 hPa and then decreased back to 100 hPa with the same step size. The fluorescent dye plumes in the water droplet were observed at each pressure for 15 s before changing the injection pressure. The total recording length was 7 minutes. During this time, the water droplet size did not change significantly due to evaporation.

In this paper, a 1.5 mm outer diameter double-barrel glass that has a theta-style cross-section (Sutter Instrument Co.) was pulled using a laser glass puller (Sutter Instrument Co., P-2000) to form theta micropipettes as shown in Figure 1b,c. Depending on the pulling parameters, the pipette tip diameter can be varied from tens of micrometers down to hundreds of nanometers. The injection channel of the theta micropipette was loaded with a saturated fluorescein (Acros Organics, Fisher Scientific; excitation/emission wavelengths 498/518 nm) aqueous solution (0.08 wt %). The extraction channel was loaded with pure water. Two plastic tubes were then inserted into the two unmodified channels at the other end of the theta capillary and sealed with epoxy (Bob Smith Ind., quick-cure 5 min epoxy). The injection channel was then connected to a source of positive pressure, while the extraction channel was connected to a source of negative pressure. In this work, the positive pressure was supplied by a pressure pump (Eppendorf, FemtoJet), and a syringe pump (New Era Pump Systems, Inc., Dual-NE-1000) was used to supply suction. To study the effects of perfusion flow on molecular diffusion around the tip of the theta pipette, the pipette tip was immersed at a 5° angle to the substrate into a large drop of water (0.3 mL) placed on a microscope slide, while fluorescent molecules where perfused through the pipette tip as illustrated in Figure 6. An inverted fluorescent microscope (Olympus FluoView FV1000 Confocal Laser Scanning Microscope; sampling speed: 2.0 µs/pixel) with a lens (LUMPLFL, 100X W NA: 1.00) placed near the bottom of the microscope slide focused on the tip end area was employed to observe the size and intensity of the fluorescent plume. A constant withdraw speed of 5 μL/min provided by the syringe pump was maintained at the probe’s extraction channel. Multiple experiments were performed at different injection channel pressures, varying from 100 hPa to 116 hPa with of 2–3 hPa increments, and then decreased back to 100 hPa with the same step size. The fluorescent dye plumes in the water droplet were observed and recorded.

### Finite element model

A 3D model was built in COMSOL Multiphysics (v4.4) to evaluate the perfusion probe’s performance (see Supporting Information File 2). Two different stationary models were developed and coupled in this model, one for the Navier–Stokes equations (Equation 2 and Equation 3) for flow parameters inside the computational region [43–44]:

[2]
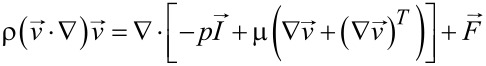


[3]
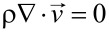


where ρ is the density, 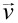 is the calculated flow velocity field, *p* is the pressure, 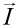 is the unit vector, μ is the dynamic viscosity, and 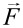 is the volume force field. Another study solved the convection diffusion equations (Equation 4 and Equation 5) for concentration distribution [45–46]:

[4]
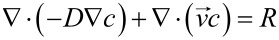


[5]
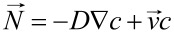


Where *D* is the diffusion coefficient, *c* is the species mass concentration, 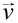 is the flow velocity field calculated from the previous study, *R* is the reaction rate, and 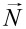 is the flux, respectively.

For hydrodynamic confinement verification, a 400 μm long quartz theta pipette was built at the top center in a water-filled rectangular computational region of (500 × 400 × 400 μm^3^). The theta pipette had a tip diameter of 20 μm, and its outer wall was formed by a truncated cone with half-angle of 8.5°. Its separation was formed by a rectangle frustum, used in the simulation to achieve a similar pipette tip geometry as for the experimental images. All the walls of this geometry were defined as no-slip walls. A negative pressure and a positive pressure was defined respectively on the top boundaries of the two channels to form the injection and extraction flow in the laminar flow module. The calculated flow field was then used as the flow parameters in the convection and diffusion study. Under our experimental conditions, diffusion was found to have practically no effect on the flow. To set up the flow conditions, a constant pressure of −120 hPa was provided at one channel of this pipette to apply suction. At the other channel, a positive pressure was applied from 100 to 117 hPa at several step increments. The upper boundary of the model is set to open boundary. These flow parameters were set to match experimental conditions. For convection and diffusion studies, the initial species concentration of the whole computational area was set to zero and the inflow concentration was set to 2407 μM (fluorescein saturated aqueous solution). The diffusion coefficient was set to 0.425 × 10^−5^ cm^2^/s (fluorescein diffusion coefficient in water at room temperature [47]). No cells or consumers were included in this hydrodynamic confinement discussion.

Then similar model parameters were used to evaluate the probe for cell oxygen consumption sensing with modifications to introducing the cell and the substrate to the model (Figures 3–5). To be specific, the rectangular computational region was reduced to (410 × 300 × 300 µm^3^) and the substrate was placed at the bottom of the calculation area. For Figure 3, the tip diameter of the pipette was set to 12 μm to have a high spatial resolution necessary for single-cell studies. An ellipsoid with 5 μm, 5 μm, and 2.5 μm related to the *a-*, *b-* and *c*-axes, respectively, was attached to the substrate to represent a cell. This ellipsoid was defined as an oxygen reactor with reaction rate of 0.04 mol/m^3^s, which resulted in a total oxygen consumption rate of 10^−17^ mol/s. The boundaries of the reactor were set to be slip so that the flow velocity does not artificially set to zero. Simulations were run for tip–substrate distances varying from 5.2 μm to 16 μm. A negative pressure and an equal value positive pressure were defined respectively on the top boundaries of the two channels to form the injection and extraction flow. The upper boundary of the model was set as open boundary. For convection and diffusion studies, the injection boundary concentration was set to 250 μM (saturated oxygen concentration in water at room temperature [48]). A symmetric boundary condition of 250 μM was set to the extraction boundary, as well as the upper boundary of the computational region. The diffusion coefficient of oxygen was set to 2 × 10^−5^ cm^2^/s (oxygen in water at room temperature [49]). The oxygen concentration difference was recorded inside the extraction channel of the theta pipette at *P* = 0, or 150 μm above the tip for Figure 3, and *P* = 150 μm for Figure 4 and Figure 5. A tetrahedral mesh with maximum mesh size of 14.4 μm, minimum mesh size of 0.615 μm, maximum element growth rate of 1.35, curvature factor of 0.3, and resolution of narrow regions of 0.85 was used to divide the system for FEM calculation. We verified that the meshes and the computational region size used here were appropriate for solving by comparing to a finer mesh setting or larger computational region. The calculated concentration differences between these two cases is less than 5%, and compared to a wider computational region setting of 410 × 320 × 320 μm^3^, the calculated concentration differences between these two cases is less than 1%. Directed solvers were selected in all studies to have the most accurate result with relative tolerance set to 10^−6^.

## Supporting Information

File 1 1D analytical model of the system.

File 2 Open theta cell.
