# Supplementary material for: Perfusion double-channel micropipette probes for oxygen flux mapping with single-cell resolution
Source: Beilstein J Nanotechnol. 2018 Mar 9;9:850–60. doi: 10.3762/bjnano.9.79 (PMC5852649; doi:10.3762/bjnano.9.79)
Supplement: File 1 — 1D analytical model of the system. [file Beilstein_J_Nanotechnol-09-850-s001.pdf]

Supporting Information 1

for

Perfusion double-channel micropipette probes for oxygen flux mapping with single cell resolution

Yang Gao[1], Bin Li[1], Riju Singhal[2], Adam Fontecchio[1], Ben Pelleg[1],

Zulfiya Orynbayeva[3]*, Yury Gogotsi[2]*, and Gary Friedman[1]*

Address: [1]Department of Electrical and Computer Engineering, Drexel University, 3141 Chestnut Street, Philadelphia, PA 19104, USA, [2]Department of Material Science and Engineering, Drexel University, 3141 Chestnut Street, Philadelphia, PA 19104, USA and [3]Department of Surgery, Drexel University, 245 N. 15th Street, Philadelphia, PA 19102, USA

Email: Zulfiya Orynbayeva - zo25@drexel.edu; Yury Gogotsi - yg36@drexel.edu;

Gary Friedman - gary@ece.drexel.edu

* Corresponding Author

1D Analytical model of the system

In this section, a simplified 1D analytical model shown in Figure S1 is constructed to demonstrate that by introducing flow velocity into the system, perfusion converts the overall consumption of oxygen of the cell into a concentration difference signal at two locations remote from the cell without mapping the oxygen gradients. To arrive at this approximate analytical model, one can replace the true 3D concentration, velocity, and consumption/production fields, by their flow cross-section averaged quantities,

while considering variation of these averaged quantities along the flow. This approach is justified when the dimension along the fluid flow is much larger than the other dimensions. In this approach, we also assume that any leakage of the consumed or produced species of interest is effectively lumped with the volumetric consumption/production rate. To be specific, as shown in Figure S1, the cell was represented as a consumption region with a width of 2*b*, centered at the origin with a consumption rate of *R*. The inlet/outlet of the flow is located symmetric to origin at distances equal to −*d* and *d*. Assume *b* is much smaller than *d*. At *t* = 0, the initial concentration of the system is *C*, a solution with known concentration of *C* is flowing from the inlet through the consumer to outlet at velocity *v*. At *x* = *d*, the flow reaches the external environment and the concentration becomes known and equal to its initial concentration. The diffusion rates are the same everywhere in the system. The convection-diffusion transportation can be described by Equation S1:

$$D\nabla^2 C - \vec{v}.\nabla C = R + \frac{\partial C}{\partial t} \qquad \text{(S1)}$$

where *C* is the molecular oxygen concentration, *D* is the oxygen diffusion coefficient (2×10^{-5} cm^2/s) in the fluid medium, $\vec{v}$ is the local fluid velocity field.

In this 1D approximation of the system shown as Figure S1, the convection-diffusion equation becomes:

$$D\frac{\partial^2}{\partial x^2}C(x,t) - \vec{v}.\nabla C(x,t) = R + \frac{\partial C(x,t)}{\partial t} \qquad \text{(S2)}$$

In the consumer region, where oxygen consumption per unit volume is *R*, we have

$$D\frac{\mathrm{d}^2 C_m(x)}{\mathrm{d}x^2} - V.\frac{\mathrm{d}C_m(x)}{\mathrm{d}x} = R \qquad \text{(S3)}$$

$C_m(x)$ – oxygen concentration in consumer range (‘m’ representing ‘middle’)

On the upstream and downstream sides, we have

$$D\frac{\mathrm{d}^2C_l(x)}{\mathrm{d}x^2} - V.\frac{\mathrm{d}C_l(x)}{\mathrm{d}x} = 0 \qquad \text{(S4a)}$$

$$D\frac{\mathrm{d}^2C_r(x)}{\mathrm{d}x^2} - V.\frac{\mathrm{d}C_r(x)}{\mathrm{d}x} = 0 \qquad \text{(S4b)}$$

$C_l(x)$ – Oxygen concentration in upstream range ('l' representing 'left')

$C_r(x)$ – Oxygen concentration in downstream range ('r' representing 'right')

The general form of the solutions (S3-S4) are:

$$C_m(x) = S_m \cdot exp\left(\frac{V}{D}x\right) + B_m - \frac{R}{V}x \qquad \text{(S4c)}$$

$$C_l(x) = S_l \cdot exp\left(\frac{V}{D}x\right) + B_l \qquad \text{(S4d)}$$

$$C_l(x) = S_r \cdot exp\left(\frac{V}{D}x\right) + B_r \qquad \text{(S4e)}$$

where constants S_m, S_l, S_r, B_m, B_l and B_r have to be determined to satisfy various boundary conditions in the 3 regions. The validity of the above solutions forms can be verified substituting them back into (S3-S4). Using the initial concentration $C(-d,t) = C_O$, the continuity of the concentration across boundaries of the consumption region $-b \le x \le b$, and the continuity of flux due to both diffusion and convection at the same boundaries, one can obtain the coefficients S_m, S_l, S_r, B_m, B_l and B_r and confirm that

$$C_O - C_r(x) = \frac{Rb}{V}\left(\frac{1 + \frac{D}{Vb}\sinh\frac{V}{D}(d-b)}{\sinh\frac{V}{D}d} - \frac{D}{Vb}exp\left(-\frac{V}{D}b\right)\right) . \left(exp\frac{V}{D}d - exp\frac{V}{D}x\right) \qquad \text{(S5)}$$

It is clear from the above solution that, when we measure the concentration difference

in this system, the consumption rate *R* can be directly inferred based on the perfusion.

When *V* is within a certain range, such that

$$\frac{V}{D}b \ll 1 \qquad \text{(S6a)}$$

$$\frac{V}{D}d \gg 1 \qquad \text{(S6b)}$$

The solution of Equation S6 can be simplified as

$$C_O - C_r(x) = \frac{Rb}{V}\left(\frac{1+\frac{D}{Vb}\left(sinh\frac{V}{D}d\; cosh\frac{V}{D}b - sinh\frac{V}{D}b\; cosh\frac{V}{D}d\right)}{sinh\frac{V}{D}d} - \frac{D}{Vb}exp\left(-\frac{V}{D}b\right)\right).\left(exp\frac{V}{D}d - exp\frac{V}{D}x\right) = -\frac{Rb}{V}\left(exp\frac{V}{D}d - exp\frac{V}{D}x\right) \qquad \text{(S7)}$$

To be specific of the range of *V* for this simplified expression, we set *D* = 10^3 µm^2/s, a typical diffusion constant for a metabolic species. In the pipette system, *b* is 5 µm, *d* = 5 cm, which is much larger than *b*. To fulfill the conditions above in Equation S7, the estimate *V* should be in the range of 0.2 µm/s to 20 µm/s. For oxygen concentration distribution problem, *D* = 2 × 10^3 µm^2/s, then *V* should be in the range of 0.4 µm/s to 40 µm/s.

From this simplified solution in this particular *V* range, the concentration distribution along *x* is only related to *V*, *D*, and *d*. For a given pipette, the *d* is fixed and for a given species, the *D* is constant inside the system. The only factor that changes the distribution is *V*. For a given flow condition setting at the in/outflow end, *V* is fixed, the amplitude of the concentration difference is proportional to *R*, the measured oxygen concentration at position *x* can reflect and track the changes of oxygen concentration of cell.

Also, it is important to see the extent to which perfusion increase the signal strength of a consumption rate *R*. Without perfusion, the solution of the convection-diffusion

equation is:

$$C_O - C_r(x) = -\frac{Rb}{D}(x-d) \qquad \text{(S8)}$$

The amplification factor due to perfusion is the ratio of the right hand side of Equation S7 to Equation S8 as

$$A = \left(\frac{exp\frac{V(d-x)}{D}-1}{\frac{V(d-x)}{D}}\right)exp\frac{V}{D}x \qquad \text{(S9)}$$

When $x \rightarrow d$, the first multiplication factor in Equation S9 $\rightarrow$ 1. Since $exp\frac{V}{D}d \leq 1$, $A \leq 1$. When x decreases, the first factor increases while the second factor decreases. When $x \rightarrow 0$, we have $exp\frac{V}{D}d > 1$, and $exp\frac{V}{D}x > 1$, so still, $A \gg 1$. It is clear that at the given flow rate range, the perfusion can amplify the concentration difference.

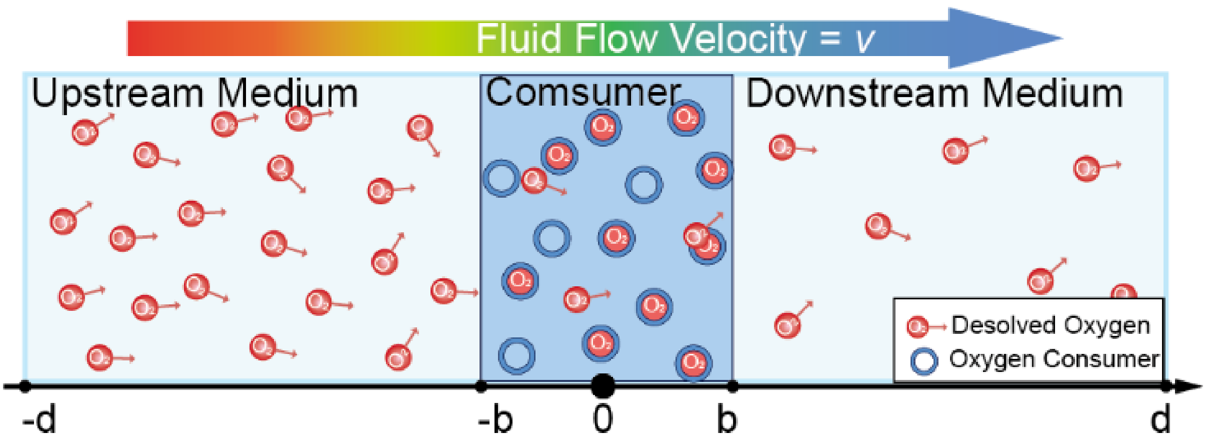

Figure S1: Illustration of the simplified analytical model of the oxygen solution, including diffusion, convection and reaction interaction inside a perfusion probe. The long arrow on top shows that the direction of flow is from the upstream to downstream medium. The red balls represent the dissolved oxygen molecules. The arrows on each oxygen molecule shows its moving direction, which is a combined result of diffusion and convection. The deep blue region with rings represents the consumer, mimicking a living cell that keeps consuming oxygen. In this region, the dissolved oxygen concentration is reduced at a certain rate.
