# Supplementary material for: Perfusion double-channel micropipette probes for oxygen flux mapping with single-cell resolution
Source: Beilstein J Nanotechnol. 2018 Mar 9;9:850–60. doi: 10.3762/bjnano.9.79 (PMC5852649; doi:10.3762/bjnano.9.79)

Supporting Information 2

for

Perfusion double-channel micro-pipette probes for oxygen flux mapping with single cell resolution

Yang Gao[1], Bin Li[1], Riju Singhal[2], Adam Fontecchio[1], Ben Pelleg[1],
Zulfiya Orynbayeva[3]*, Yury Gogotsi[2]*, and Gary Friedman[1]*

Address: [1]Department of Electrical and Computer Engineering, Drexel University, 3141 Chestnut Street, Philadelphia, PA 19104, USA, [2]Department of Material Science and Engineering, Drexel University, 3141 Chestnut Street, Philadelphia, PA 19104, USA and [3]Department of Surgery, Drexel University, 245 N. 15th Street, Philadelphia, PA 19102, USA

Email: Zulfiya Orynbayeva - zo25@drexel.edu; Yury Gogotsi - yg36@drexel.edu;
Gary Friedman - gary@ece.drexel.edu
* Corresponding Author

COMSOL MULTIPHYSICS®

Open theta cell

Contents

1. Global 3
   1.1. Definitions 3
2. Model 1 4
   2.1. Definitions 4
   2.2. Geometry 1 6
   2.3. Materials 7
   2.4. Laminar Flow 8
   2.5. Transport of Diluted Species 10
   2.6. Mesh 1 11
3. Study 1 12
   3.1. Parametric Sweep 12
   3.2. Parametric Sweep 2 12
   3.3. Stationary 12
   3.4. Stationary 2 12
   3.5. Time Dependent 2 13
   3.6. Stationary 3 13
   3.7. Stationary 4 14
4. Results 15
   4.1. Data Sets 15
   4.2. Derived Values 25
   4.3. Tables 28
   4.4. Plot Groups 30

1 Global

Global settings

Name	1210 to 0115 open theta cell408.mph
Path	D:\YangGao\1210 to 0115 open theta cell408.mph
Program	COMSOL 4.4 (Build: 150)

Used products

COMSOL Multiphysics
CAD Import Module
CFD Module

1.1 Definitions

1.1.1 Parameters 1

Parameters

Name	Expression	Value	Description
r1	60	60.000	
x1	0	0.0000	
p1	1	1.0000	

2 Model 1

2.1 Definitions

2.1.1 Probes

Domain Point Probe 2

Probe type	Domain point probe

Domain Probe 1

Probe type	Domain probe

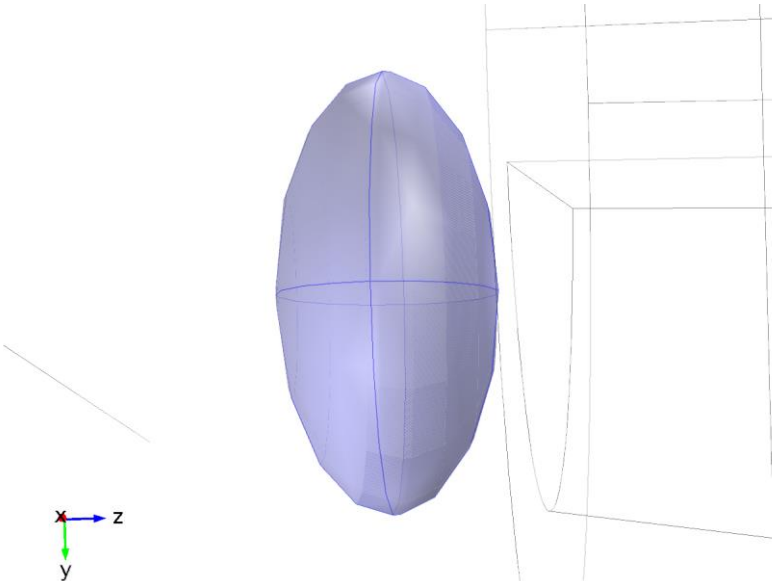

Selection

Boundary Probe 3

Probe type	Boundary probe

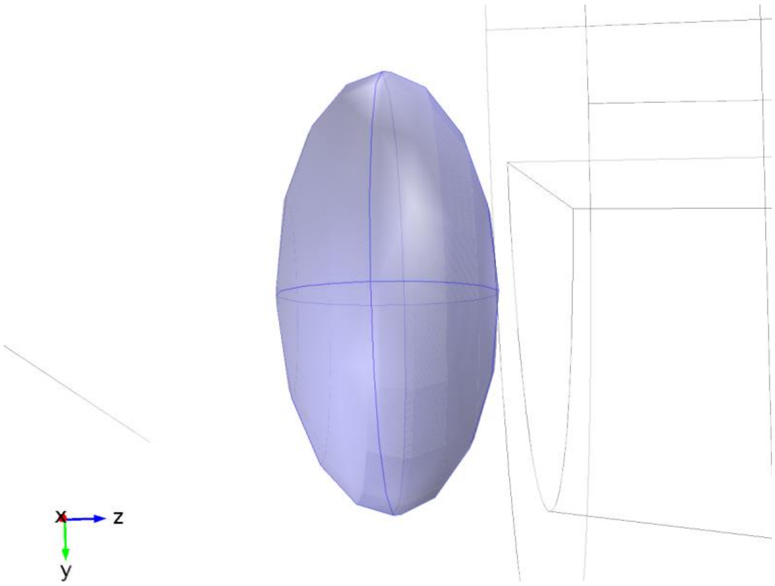

Selection

Boundary Probe 4

Probe type	Boundary probe

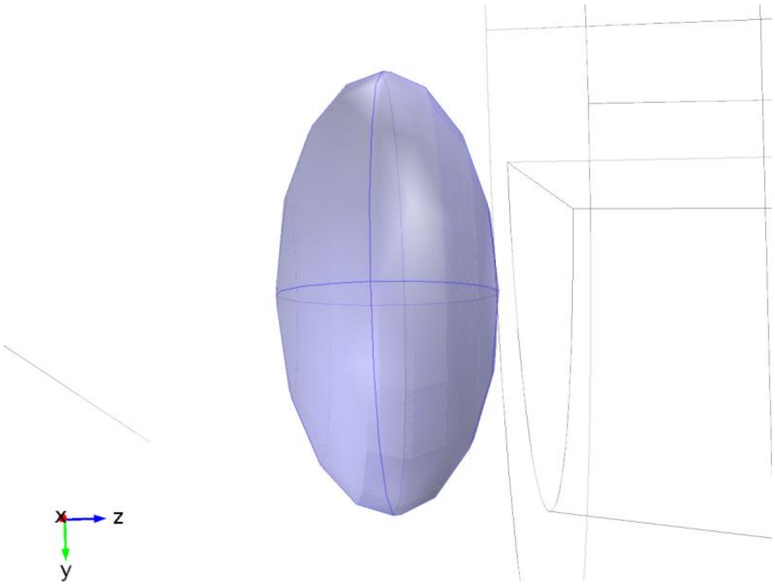

Selection

Domain Point Probe 5

Probe type	Domain point probe

2.1.2 Selections

Explicit 1

Selection type
Explicit

Selection
No edges

2.1.3 Coordinate Systems

Boundary System 1

Coordinate system type	Boundary system
Tag	sys1

2.2 Geometry 1

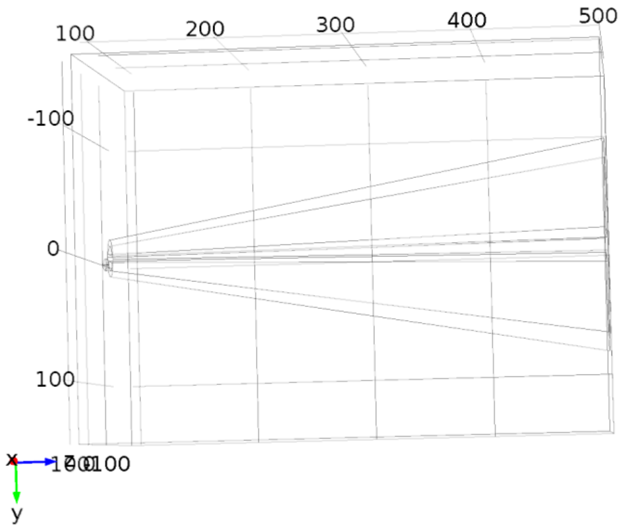

Geometry 1

Units

Length unit	µm
Angular unit	deg

2.3 Materials

2.3.1 Water

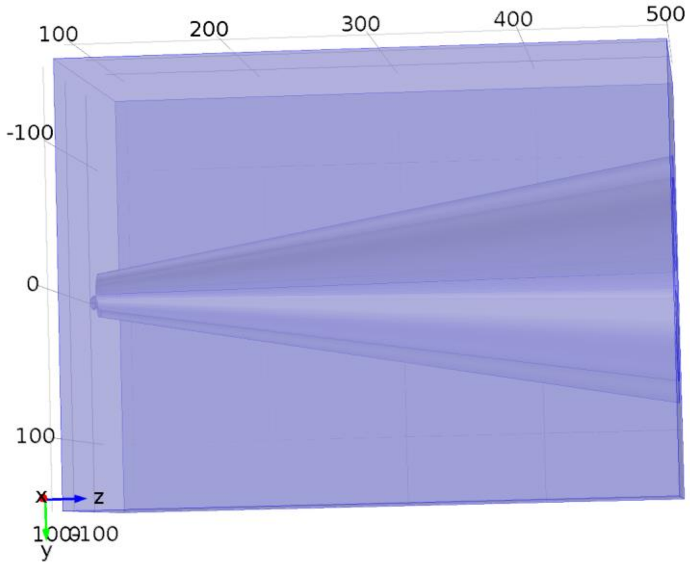

Water

Selection

Geometric entity level	Domain
Selection	Domains 1, 3–5

2.3.2 Silica glass

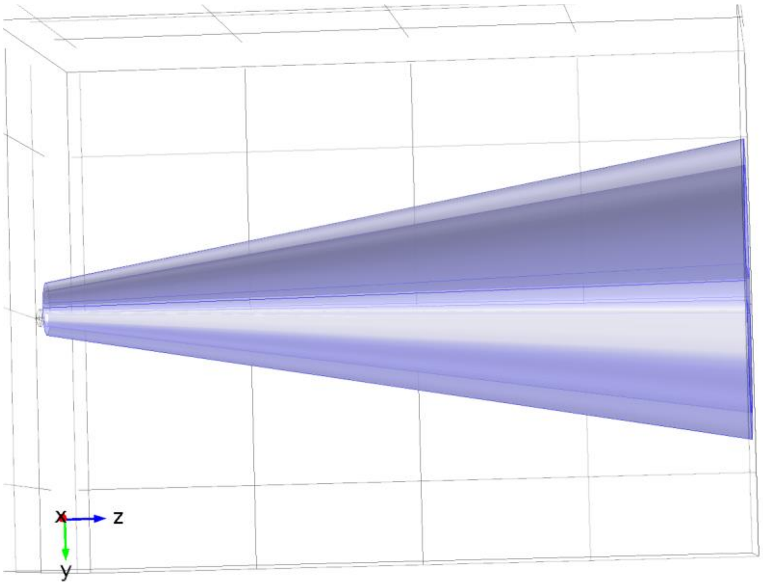

Silica glass

Selection

Geometric entity level	Domain
Selection	Domain 2

2.4 Laminar Flow

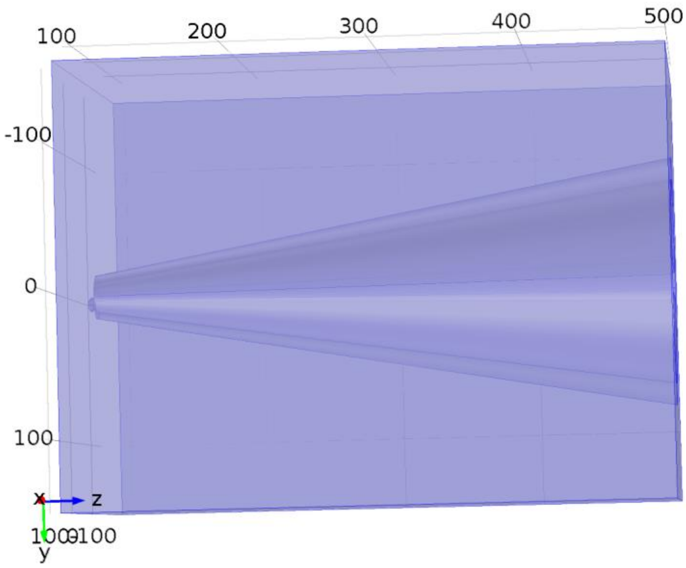

Laminar Flow

Equations

$$\rho(\mathbf{u}\cdot\nabla)\mathbf{u} =$$
$$\nabla\cdot\left[-p\mathbf{I}+\mu\left(\nabla\mathbf{u}+(\nabla\mathbf{u})^{T}\right)\right]+\mathbf{F}$$
$$\rho\nabla\cdot(\mathbf{u})=0$$

Features

Fluid Properties 1
Wall 1
Initial Values 1
Inlet 1
Outlet 1
Interior Wall 1

2.4.1 Fluid Properties 1

Equations

$$\rho(\mathbf{u}\cdot\nabla)\mathbf{u} =$$
$$\nabla\cdot\left[-p\mathbf{I}+\mu\left(\nabla\mathbf{u}+(\nabla\mathbf{u})^T\right)\right]+\mathbf{F}$$
$$\rho\nabla\cdot(\mathbf{u})=0$$

2.4.2 Wall 1

Equations

$$\mathbf{u}=\mathbf{0}$$

2.4.3 Inlet 1

Equations

$$p=p_0,\ \left[\mu\left(\nabla\mathbf{u}+(\nabla\mathbf{u})^T\right)\right]\mathbf{n}=0$$

2.4.4 Outlet 1

Equations

$$p=p_0,\ \left[\mu\left(\nabla\mathbf{u}+(\nabla\mathbf{u})^T\right)\right]\mathbf{n}=0$$

2.4.5 Interior Wall 1

Equations

$$\mathbf{u}_u\cdot\mathbf{n}=0$$
$$\mathbf{u}_d\cdot\mathbf{n}=0$$
$$\mathbf{K}_u-(\mathbf{K}_u\cdot\mathbf{n})\mathbf{n}=\mathbf{0},\ \mathbf{K}_u=\left[\mu\left(\nabla\mathbf{u}+(\nabla\mathbf{u})^T\right)\right]_u(-\mathbf{n})$$
$$\mathbf{K}_d-(\mathbf{K}_d\cdot\mathbf{n})\mathbf{n}=\mathbf{0},\ \mathbf{K}_d=\left[\mu\left(\nabla\mathbf{u}+(\nabla\mathbf{u})^T\right)\right]_d\mathbf{n}$$

2.5 Transport of Diluted Species

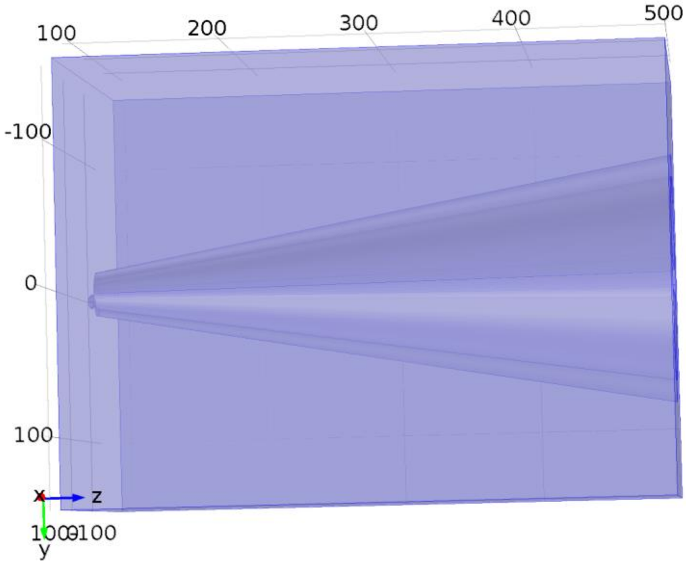

Transport of Diluted Species

Equations

$$\nabla \cdot (-D_i \nabla c_i) + \mathbf{u} \cdot \nabla c_i = R_i$$
$$\mathbf{N}_i = -D_i \nabla c_i + \mathbf{u} c_i$$

Features

Convection and Diffusion 1
No Flux 1
Initial Values 1
Inflow 1
Outflow 1
Concentration 1
Reactions 1
Initial Values 2

2.5.1 Convection and Diffusion 1

Equations

$$\nabla \cdot (-D_i \nabla c_i) + \mathbf{u} \cdot \nabla c_i = R_i$$
$$\mathbf{N}_i = -D_i \nabla c_i + \mathbf{u} c_i$$

2.5.2 No Flux 1

Equations

$$-\mathbf{n}\cdot\mathbf{N}_i=0$$

2.5.3 Inflow 1

Equations

$$c_i=c_{0,i}$$

2.5.4 Outflow 1

Equations

$$-\mathbf{n}\cdot D_i\nabla c_i=0$$

2.5.5 Concentration 1

Equations

$$c_i=c_{0,i}$$

2.5.6 Reactions 1

Equations

$$\nabla\cdot(-D_i\nabla c_i)+\mathbf{u}\cdot\nabla c_i=R_i$$

2.6 Mesh 1

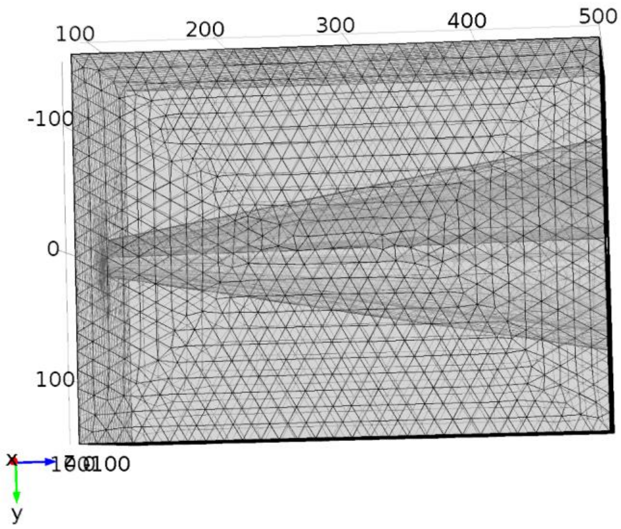

Mesh 1

3 Study 1

3.1 Parametric Sweep

Parameter name	Parameter value list
r1	range(20,10,60)

3.2 Parametric Sweep 2

Parameter name	Parameter value list
x1	range(-90,10,90)

3.3 Stationary

Study settings

Description	Value
Include geometric nonlinearity	Off

Physics and variables selection

Physics interface	Discretization
Laminar Flow (spf)	physics

Mesh selection

Geometry	Mesh
Geometry 1 (geom1)	mesh1

3.4 Stationary 2

Study settings

Description	Value
Include geometric nonlinearity	Off

Physics and variables selection

Physics interface	Discretization
Laminar Flow (spf)	physics
Transport of Diluted Species (chds)	physics

Mesh selection

Geometry	Mesh
Geometry 1 (geom1)	mesh1

3.5 Time Dependent 2

Study settings

Description	Value
Include geometric nonlinearity	Off

Times	Unit
range(0,5,3000)	s

Physics and variables selection

Physics interface	Discretization
Laminar Flow (spf)	physics
Transport of Diluted Species (chds)	physics

Mesh selection

Geometry	Mesh
Geometry 1 (geom1)	mesh1

3.6 Stationary 3

Study settings

Description	Value
Include geometric nonlinearity	Off

Physics and variables selection

Physics interface	Discretization
Laminar Flow (spf)	physics

Mesh selection

Geometry	Mesh
Geometry 1 (geom1)	mesh1

3.7 Stationary 4

Study settings

Description	Value
Include geometric nonlinearity	Off

Physics and variables selection

Physics interface	Discretization
Laminar Flow (spf)	physics
Transport of Diluted Species (chds)	physics

Mesh selection

Geometry	Mesh
Geometry 1 (geom1)	mesh1

4 Results

4.1 Data Sets

4.1.1 Solution 1

Solution

Description	Value
Solution	Solver 1
Component	Save Point Geometry 1

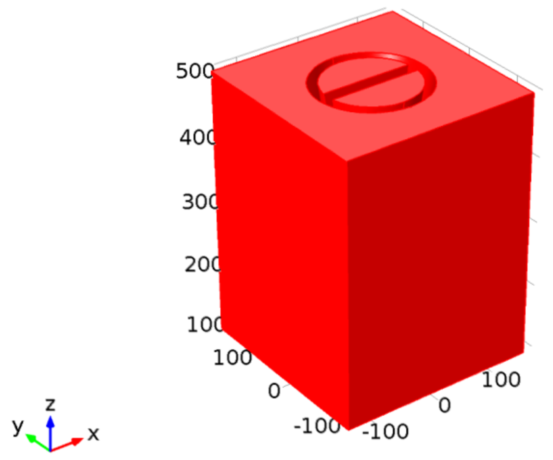

Data set: Solution 1

4.1.2 Surface 1

Data

Description	Value
Data set	Solution 1

Parameterization

Description	Value
x- and y-axes	Surface parameters

4.1.3 Surface 2

Data

Description	Value
Data set	Solution 1

Parameterization

Description	Value
x- and y-axes	Surface parameters

4.1.4 Probe Solution 2

Solution

Description	Value
Solution	Solver 1
Component	Save Point Geometry 1

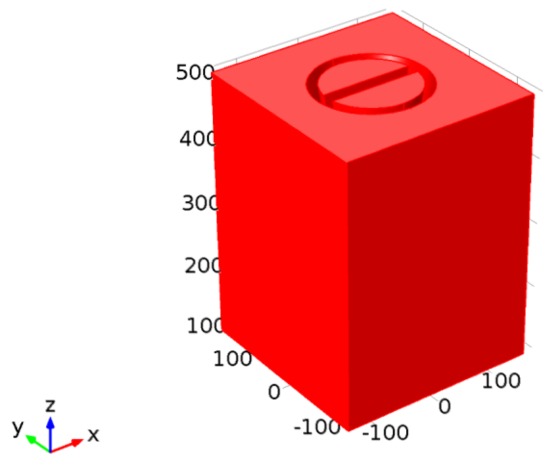

Data set: Probe Solution 2

4.1.5 Probe Solution 3

Solution

Description	Value
Solution	Solver 1
Component	Save Point Geometry 1
Frame	Spatial (x, y, z)

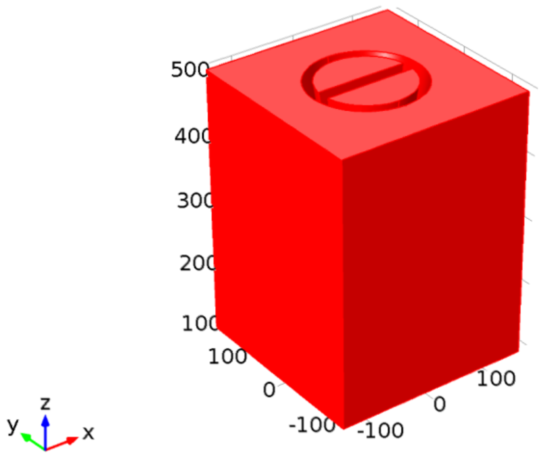

Data set: Probe Solution 3

4.1.6 Domain Point Probe 1

Data

Description	Value
Data set	Probe Solution 3

Point data

Description	Value
Entry method	Coordinates

Settings

Description	Value
x	0
y	-15
z	250

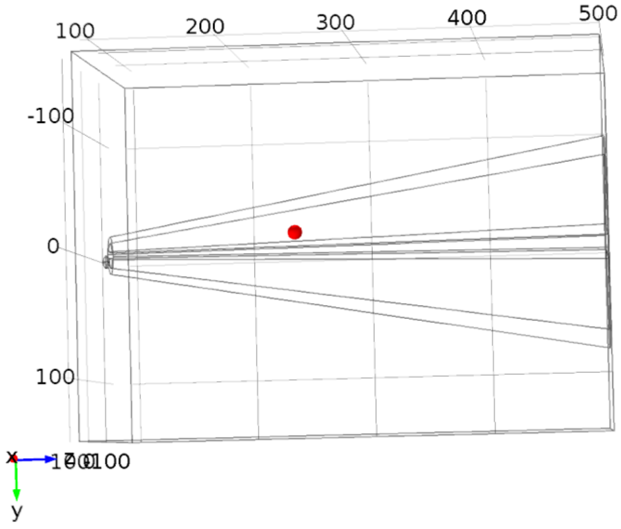

Data set: Domain Point Probe 1

4.1.7 Domain Point Probe 2

Data

Description	Value
Data set	Probe Solution 3

Point data

Description	Value
Entry method	Coordinates

Settings

Description	Value
x	0
y	15
z	250

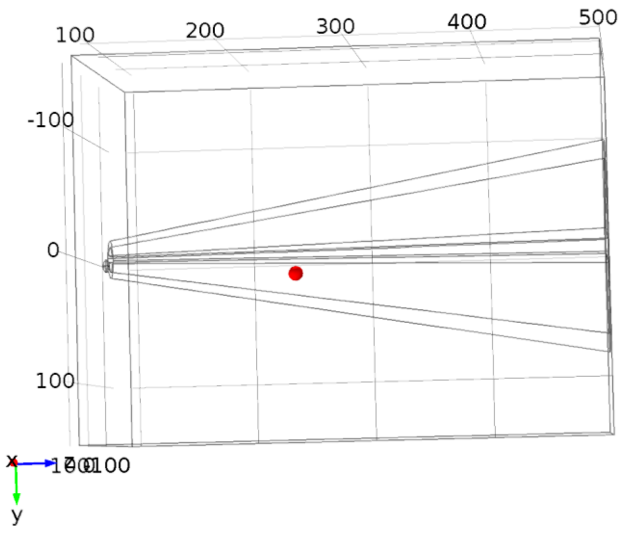

Data set: Domain Point Probe 2

4.1.8 Boundary Probe 1

Selection

Geometric entity level	Boundary
Selection	Boundaries 18–21, 24–27, 30

Data

Description	Value
Data set	Probe Solution 4

Settings

Description	Value
Method	Integration
Integration order	4
Integration order	On

4.1.9 Domain Point Probe 3

Data

Description	Value
Data set	Probe Solution 3

Point data

Description	Value
Entry method	Coordinates

Settings

Description	Value
x	0
y	10
z	150

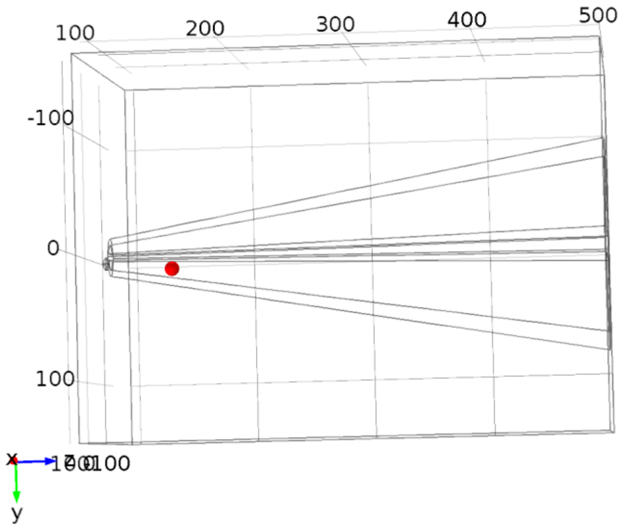

Data set: Domain Point Probe 3

4.1.10 Domain Point Probe 4

Data

Description	Value
Data set	Probe Solution 3

Point data

Description	Value
Entry method	Coordinates

Settings

Description	Value
x	0
y	12
z	200

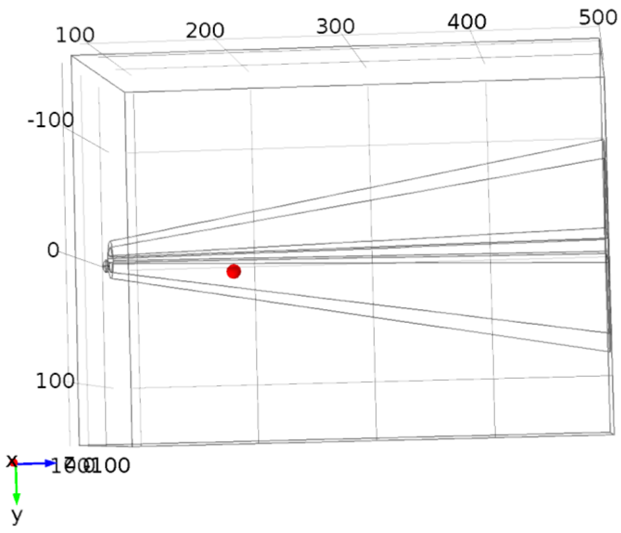

Data set: Domain Point Probe 4

4.1.11 Probe Solution 4

Solution

Description	Value
Solution	Solver 1
Component	Save Point Geometry 1
Frame	Geometry (x, y, z)

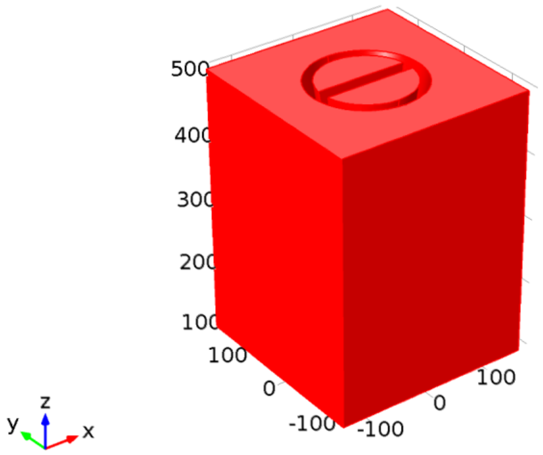

Data set: Probe Solution 4

4.1.12 Boundary Probe 2

Selection

Geometric entity level	Boundary
Selection	Boundary 16

Data

Description	Value
Data set	Probe Solution 4

Settings

Description	Value
Method	Integration
Integration order	4
Integration order	On

4.1.13 Domain Probe 1

Selection

Geometric entity level	Domain
Selection	Domain 5

Data

Description	Value
Data set	Probe Solution 2

Settings

Description	Value
Method	Integration
Integration order	4
Integration order	On

4.1.14 Boundary Probe 3

Selection

Geometric entity level	Boundary
Selection	Boundaries 18–21, 24–27

Data

Description	Value
Data set	Probe Solution 4

Settings

Description	Value
Element refinement	4

4.1.15 Boundary Probe 4

Selection

Geometric entity level	Boundary
Selection	Boundaries 18–21, 24–27

Data

Description	Value
Data set	Probe Solution 4

Settings

Description	Value
Method	Integration
Integration order	4
Integration order	On

4.1.16 Domain Point Probe 5

Data

Description	Value
Data set	Probe Solution 3

Point data

Description	Value
Entry method	Coordinates

Settings

Description	Value
x	0
y	6
z	100

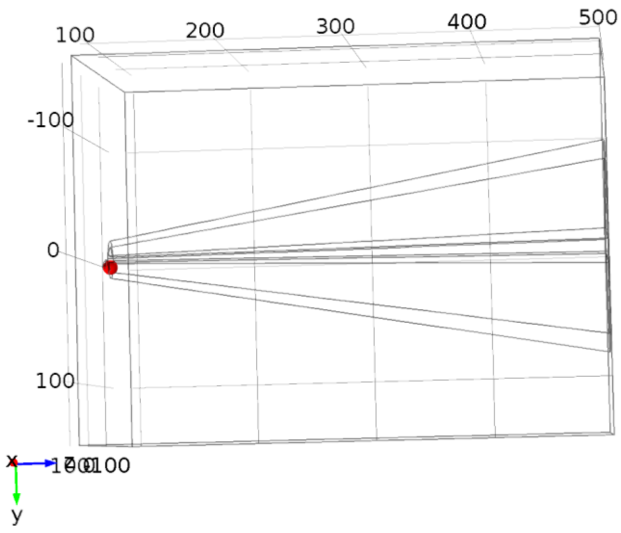

Data set: Domain Point Probe 5

4.1.17 Domain Point Probe 6

Data

Description	Value
Data set	Probe Solution 3

Point data

Description	Value
Entry method	Coordinates

Settings

Description	Value
x	0
y	7
z	110

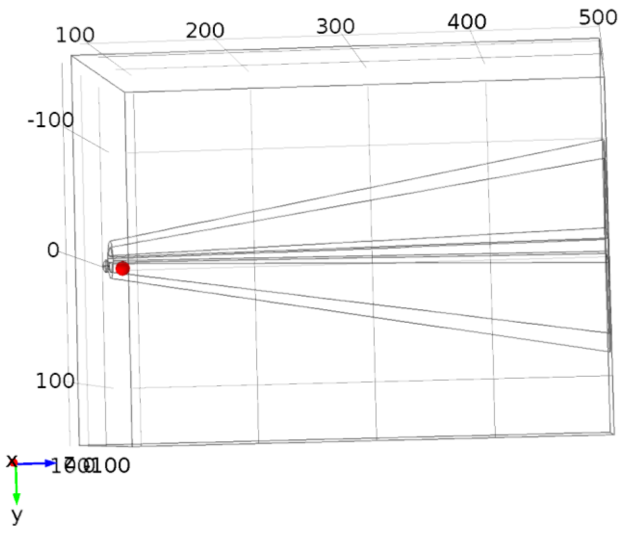

Data set: Domain Point Probe 6

4.2 Derived Values

4.2.1 Point Probe Expression 1

Data

Description	Value
Data set	Domain Point Probe 1

Expression

Description	Value
Expression	c
Unit	mmol/m^3
Description	Concentration

4.2.2 Point Probe Expression 2

Data

Description	Value
Data set	Domain Point Probe 2

Expression

Description	Value
Expression	c
Unit	mmol/m^3
Description	Concentration

4.2.3 Boundary Probe 1

Data

Description	Value
Data set	Boundary Probe 1

Expression

Description	Value
Expression	chds.ntflux_c
Unit	mol/s
Description	Normal total flux

4.2.4 Point Probe Expression 3

Data

Description	Value
Data set	Domain Point Probe 3

Expression

Description	Value
Expression	c
Unit	mmol/m^3
Description	Concentration

4.2.5 Point Probe Expression 4

Data

Description	Value
Data set	Domain Point Probe 4

Expression

Description	Value
Expression	c
Unit	mmol/m^3
Description	Concentration

4.2.6 Boundary Probe 2

Data

Description	Value
Data set	Boundary Probe 2

Expression

Description	Value
Expression	chds.ntflux_c
Unit	mol/s
Description	Normal total flux

4.2.7 Domain Probe 1

Data

Description	Value
Data set	Domain Probe 1

Expression

Description	Value
Expression	chds.R_c
Unit	mol/s
Description	Total rate expression

4.2.8 Boundary Probe 3

Data

Description	Value
Data set	Boundary Probe 3

Expression

Description	Value
Expression	spf.U
Unit	µm/s
Description	Velocity magnitude

4.2.9 Boundary Probe 4

Data

Description	Value
Data set	Boundary Probe 4

Expression

Description	Value
Expression	spf.U
Unit	µm/s
Description	Velocity magnitude

4.2.10 Point Probe Expression 5

Data

Description	Value
Data set	Domain Point Probe 5

Expression

Description	Value
Expression	c
Unit	mmol/m^3
Description	Concentration

4.2.11 Point Probe Expression 6

Data

Description	Value
Data set	Domain Point Probe 6

Expression

Description	Value
Expression	c
Unit	mmol/m^3
Description	Concentration

4.3 Tables

4.3.1 Evaluation 3D

Interactive 3D values

Evaluation 3D

x	y	z	Value
-5.6843E-14	-15.470	323.45	0.49763
5.6843E-14	-17.978	315.92	0.49976
-5.6843E-14	-17.978	320.10	0.49976
1.7053E-13	-19.651	327.63	0.49976
5.6843E-14	-17.978	318.43	0.49976
5.6843E-14	-17.142	310.07	0.49976
-5.6843E-14	-15.470	328.47	0.49977
5.6843E-14	-15.470	324.28	0.49977
1.7053E-13	-15.470	327.63	0.49977
5.6843E-14	-2.9267	91.821	0.49483
-5.6843E-14	-17.142	312.58	0.49762

x	y	z	Value
-5.6843E-14	-16.306	297.53	0.49761
-5.6843E-14	-14.633	330.14	0.49765
-5.6843E-14	-14.633	300.03	0.49761
5.6843E-14	-19.531	351.42	0.49977
2.8422E-14	-7.3883	97.769	136.84

4.3.2 Probe Table 2

Probe Table 2

p1	Concentration (mmol/m^3), Point Probe Expression 2	Total rate expression (mol/s), Domain Probe 1	Velocity magnitude (µm/s), Boundary Probe 3	Velocity magnitude (µm/s), Boundary Probe 4	Concentration (mmol/m^3), Point Probe Expression 5
0.0000	249.93	-1.0335E-17	0.0000	0.0000	249.55
0.50000	249.87	-1.0335E-17	19.377	6.4461	249.55
1.0000	249.82	-1.0335E-17	38.754	12.892	249.57
1.5000	249.79	-1.0335E-17	58.131	19.338	249.60
2.0000	249.77	-1.0335E-17	77.508	25.784	249.63
2.5000	249.77	-1.0335E-17	96.885	32.230	249.65
3.0000	249.77	-1.0335E-17	116.26	38.676	249.67
3.5000	249.78	-1.0335E-17	135.64	45.122	249.69
4.0000	249.79	-1.0335E-17	155.01	51.568	249.70
4.5000	249.80	-1.0335E-17	174.39	58.014	249.72
5.0000	249.80	-1.0335E-17	193.77	64.460	249.73
5.5000	249.81	-1.0335E-17	213.14	70.906	249.74
6.0000	249.82	-1.0335E-17	232.52	77.352	249.75
6.5000	249.83	-1.0335E-17	251.90	83.798	249.76
7.0000	249.84	-1.0335E-17	271.27	90.244	249.77
7.5000	249.85	-1.0335E-17	290.65	96.690	249.78
8.0000	249.85	-1.0335E-17	310.02	103.14	249.78
8.5000	249.86	-1.0335E-17	329.40	109.58	249.79
9.0000	249.87	-1.0335E-17	348.78	116.03	249.79
9.5000	249.87	-1.0335E-17	368.15	122.47	249.80
10.000	249.88	-1.0335E-17	387.53	128.92	249.81
10.500	249.88	-1.0335E-17	406.90	135.37	249.81
11.000	249.89	-1.0335E-17	426.28	141.81	249.81

p1	Concentration (mmol/m^3), Point Probe Expression 2	Total rate expression (mol/s), Domain Probe 1	Velocity magnitude (µm/s), Boundary Probe 3	Velocity magnitude (µm/s), Boundary Probe 4	Concentration (mmol/m^3), Point Probe Expression 5
11.500	249.89	-1.0335E-17	445.65	148.26	249.82
12.000	249.90	-1.0335E-17	465.03	154.70	249.82
12.500	249.90	-1.0335E-17	484.41	161.15	249.83
13.000	249.90	-1.0335E-17	503.78	167.59	249.83
13.500	249.91	-1.0335E-17	523.16	174.04	249.83
14.000	249.91	-1.0335E-17	542.53	180.49	249.84
14.500	249.91	-1.0335E-17	561.91	186.93	249.84
15.000	249.91	-1.0335E-17	581.28	193.38	249.84

4.4 Plot Groups

4.4.1 Velocity (spf)

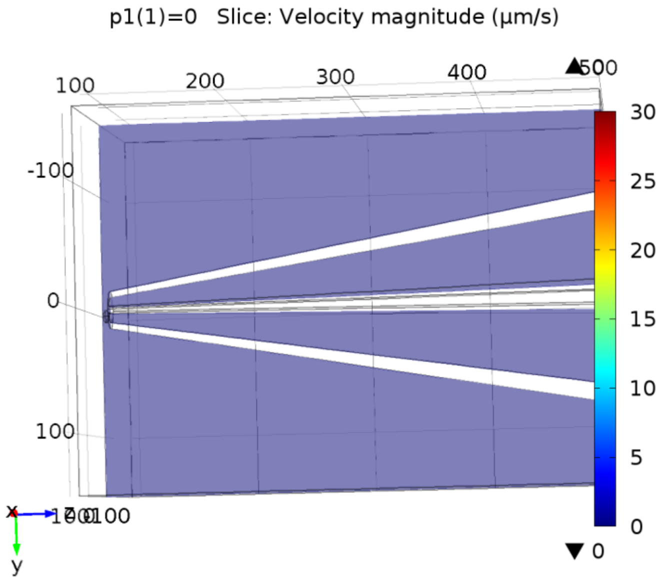

p1(1)=0 Slice: Velocity magnitude (µm/s)

4.4.2 Pressure (spf)

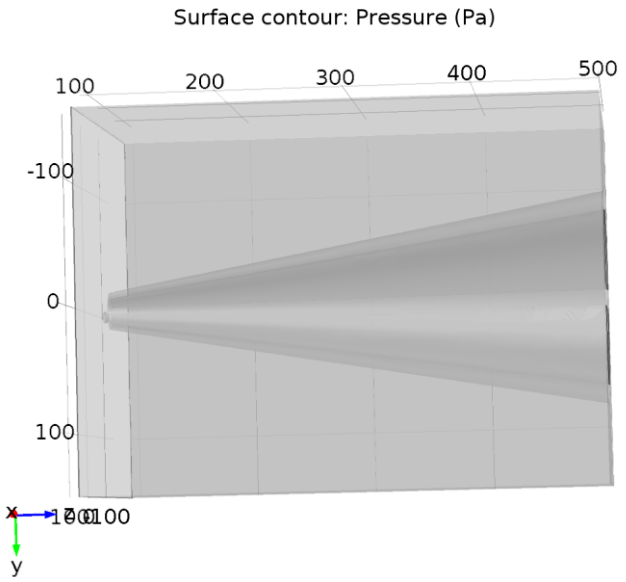

Surface contour: Pressure (Pa)

4.4.3 Concentration (chds)

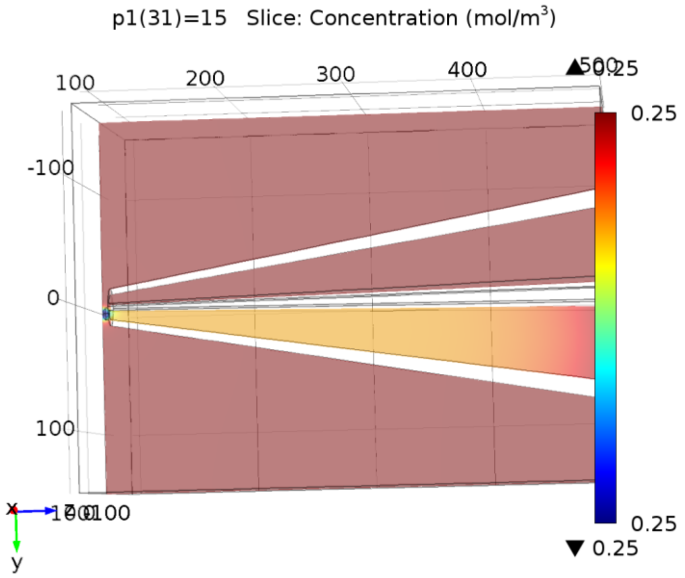

p1(31)=15 Slice: Concentration (mol/m³)

4.4.4 Concentration (chds) 1

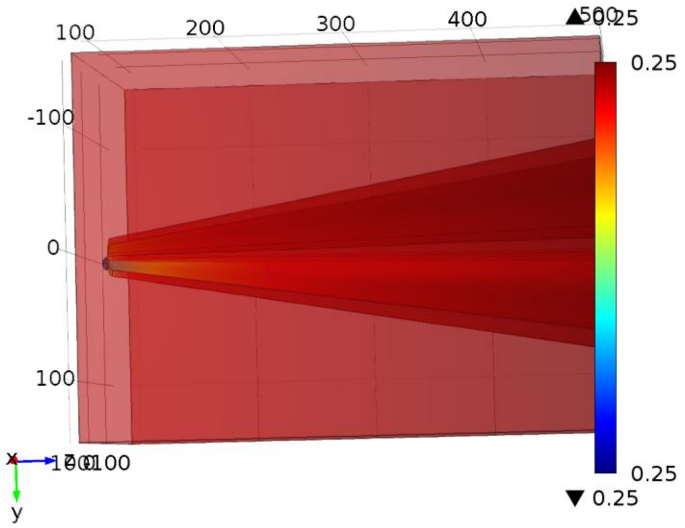

p1(1)=0 Surface: Concentration (mol/m^3)

4.4.5 Probe 1D Plot Group 17

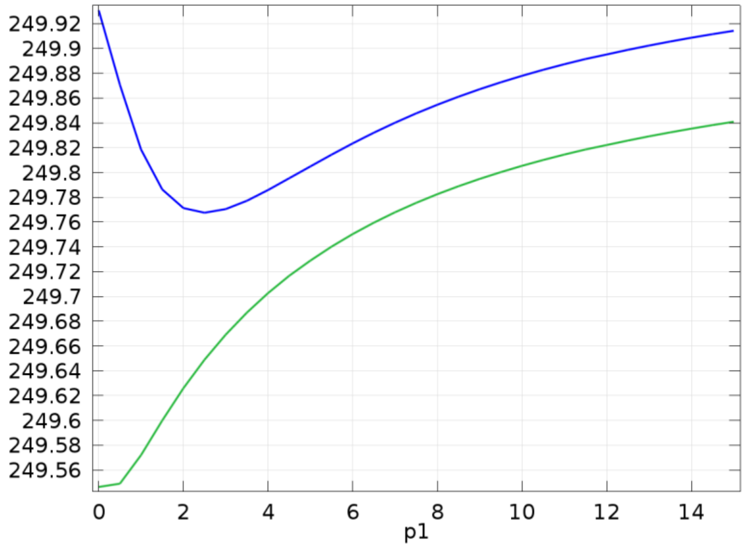

4.4.6 Probe 1D Plot Group 18

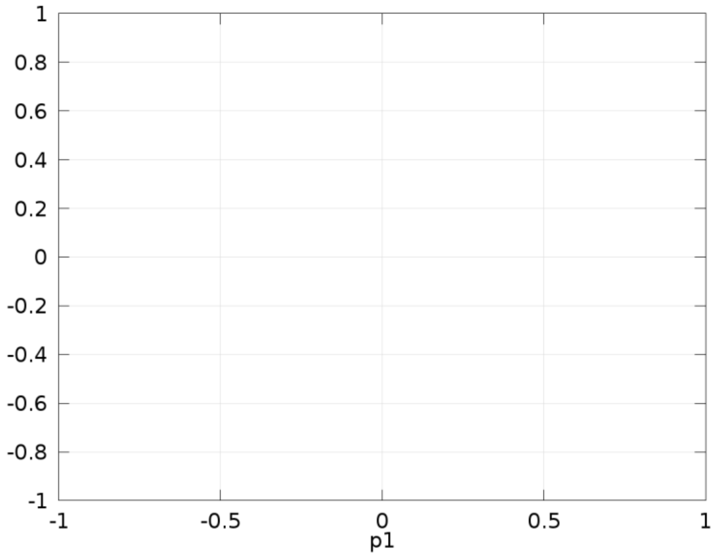

4.4.7 Probe 1D Plot Group 19

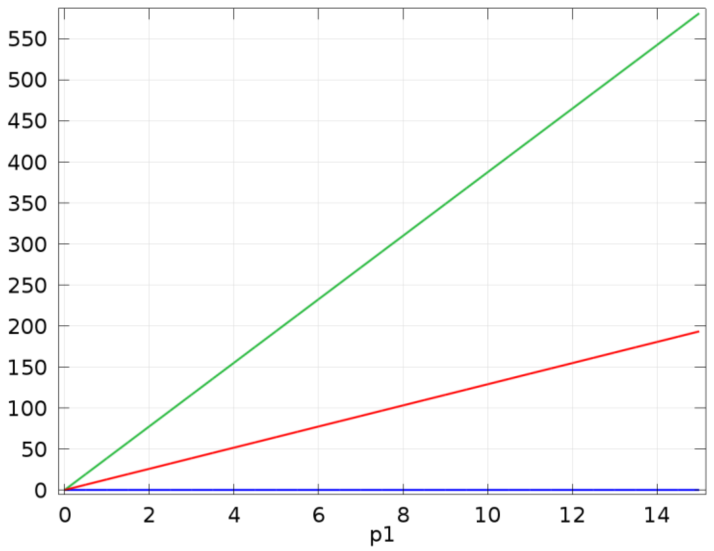

Supplement: File 2 — Open theta cell. [file Beilstein_J_Nanotechnol-09-850-s002.pdf]
